# Characterization of two GH10 enzymes with ability to hydrolyze pretreated *Sorghum bicolor* bagasse

**DOI:** 10.1007/s00253-025-13484-4

**Published:** 2025-04-28

**Authors:** Camila Bruno Baron, María Laura Mon, Rubén Marrero Díaz de Villegas, Andrea Cattaneo, Paola Di Donato, Annarita Poli, Maria Emilia Negri, Mariana Alegre, Marcelo A. Soria, María Cecilia Rojo, Mariana Combina, Ilaria Finore, Paola M. Talia

**Affiliations:** 1 Instituto de Agrobiotecnología y Biología Molecular (IABIMO), UEDD INTA-CONICET, Hurlingham, Buenos Aires Argentina; 2 Departamento de Biodiversidad y Biología Experimental, Facultad de Ciencias Exactas y Naturales, Universidad de Buenos Aires, Ciudad Autónoma de Buenos Aires, Argentina; 3 Institute of Biomolecular Chemistry (ICB), Consiglio Nazionale Delle Ricerche (CNR), Pozzuoli, Italy; 4 Department of Science and Technology, University of Naples “Parthenope”, Naples, Italy; 5 Estación Experimental Agropecuaria Pergamino, Instituto Nacional de Tecnología Agropecuaria (INTA), Pergamino, Buenos Aires Argentina; 6 Escuela de Ciencias Agrarias y Ambientales-Universidad Nacional del Noroeste de La Provincia de Buenos Aires, Pergamino, Buenos Aires Argentina; 7 Cátedra de Microbiología Agrícola, Facultad de Agronomía, Universidad de Buenos Aires, INBA UBA-CONICET, Ciudad Autónoma de Buenos Aires, Argentina; 8 Estación Experimental Agropecuaria Mendoza, Instituto Nacional de Tecnología Agropecuaria (INTA), Luján de Cuyo, Mendoza Argentina; 9 Consejo Nacional de Investigaciones Científicas y Tecnológicas (CONICET), Ciudad Autónoma de Buenos Aires, Argentina

**Keywords:** Xylanase, Bifunctional xylanase/β-glucanase, GH10, Pretreated *Sorghum bicolor* bagasse, Antioxidant activity of xylo-oligosaccharides

## Abstract

**Abstract:**

In this study, we characterized two novel enzymes of the glycoside hydrolase family 10 (GH10), Xyl10 C and Xyl10E, identified in the termite gut microbiome. The activities of both enzymes were assayed using beechwood xylan, barley β-glucan, and pretreated *Sorghum bicolor* bagasse (SBB) as substrates. Both enzymes, assessed individually and in combination, showed activity on beechwood xylan and pretreated SBB, whereas Xyl10E also showed activity on barley β-glucan. The composition of pretreated SBB mainly consisted of xylose and arabinose content. Purified Xyl10 C showed optimum xylanase activity in the pH range 7.0–8.0 and at a temperature of 50–60 °C, while Xyl10E was active at a wider pH range (5.0–10.0) and at 50 °C. The residual activities of Xyl10 C and Xyl10E after 8 h of incubation at 40 °C were 85% and 70%, respectively. The enzymatic activity of Xyl10 C increased to 115% in the presence of 5 M NaCl, was only inhibited in the presence of 0.5% sodium dodecyl sulfate (SDS), and decreased with β-mercaptoethanol. The xylanase and glucanase activities of Xyl10E were inhibited only in the presence of MnSO_4_, NaCl, and SDS. The main hydrolysis enzymatic product of Xyl10 C and Xyl10E on pretreated SBB was xylobiose. In addition, the xylo-oligosaccharides produced by xylanase Xyl10E on pretreated SBB demonstrated promising antioxidant activity. Thus, the hydrolysis products using Xyl10E on pretreated SBB indicate potential for antioxidant activity and other valuable industrial applications.

**Key points:**

• *Two novel GH10 xylanases from the termite gut microbiome were characterized.*

• *Xylo-oligosaccharides obtained from sorghum bagasse exhibited antioxidant potential.*

• *Both enzymes and their hydrolysis product have potential to add value to agro-waste.*

**Graphical Abstract:**

**Figure:**
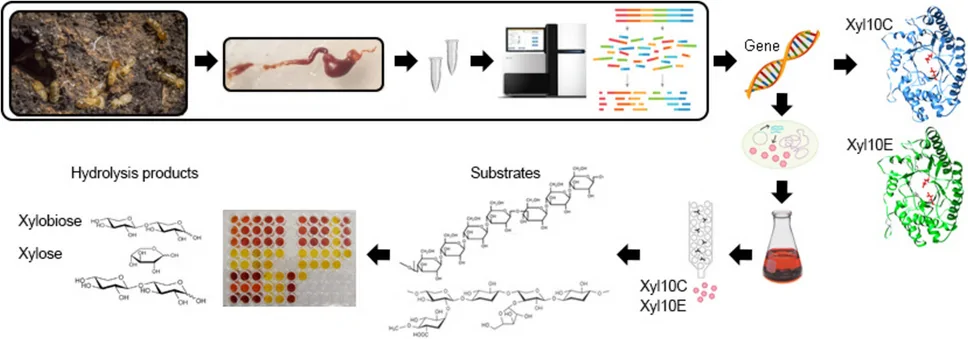

**Supplementary Information:**

The online version contains supplementary material available at 10.1007/s00253-025-13484-4.

## Introduction

Lignocellulose, which is mainly composed of cellulose, hemicellulose, and lignin, is the most abundant and renewable source of carbon on Earth. However, lignocellulosic biomass from plants is recalcitrant due to its complex polymer composition (Adegboye et al. 2021; Meenakshisundaram et al. 2021). For greater recovery of the sugar components of polysaccharides from lignocellulosic biomass, physical, chemical, and/or biological pretreatment is generally performed before enzymatic hydrolysis. To partially obtain hemicellulose and remove lignin, a basic chemical pretreatment is the most suitable and used method (Basak et al. 2023; Chen et al. 2017; Saini et al. 2015). However, the costs associated with this process are still high, which hinders its large-scale commercialization (Adegboye et al. 2021).

Some studies evaluating raw materials for industrial applications have investigated the potency of sweet sorghum (*Sorghum bicolor*) bagasse (Bagewadi et al. 2016; Castro et al. 2021; Nunta et al. 2023; Wei et al. 2018), which is the residue remaining after extraction of the stem juice and represents approximately 36% of the plant (Pengilly et al. 2015). In addition, sorghum is considered a promising energy crop for use in biofuels and as a source of value-added biomolecules due to its high photosynthetic rate, great genetic diversity, and drought resistance (Barcelos et al. 2016; Bonin et al. 2016).

Enzymes, including xylanases and glucanases, play a fundamental role in the transformation of the traditional chemical industry into a more sustainable and environmentally friendly alternative (Talens-Perales et al. 2021). Xylanases (EC 3.2.1.8) catalyze the hydrolysis of the internal 1,4-β-D xylosidic linkages in xylan, the most abundant polysaccharide in hemicellulose in nature. Xylanases occur in several glycosyl hydrolase (GH) families of the Carbohydrate-Active enZYmes (CAZy) database classification, including GH5, GH8, GH10, GH11, GH30, GH43, and GH141. In particular, the GH10 and GH11 families are considered true xylanases because of their substrate specificity (Paës et al. 2012; Talens-Perales et al. 2021). Xylanases are widely used across various industries, including pulp and paper production, juice clarification, enhancement of animal digestibility, and production of second-generation bioethanol (Talens-Perales et al. 2021). More recently, xylanases have been used in the production of xylo-oligosaccharides (XOs), mainly xylobiose to be used as prebiotics, and offers a range of biological benefits, such as antioxidant and antimicrobial effects, among others (Chen et al. 2021; Zarafeta et al. 2020).

Glucanases, on the other hand, are able to hydrolyze β-glucan into cello-oligosaccharides (COs) and glucose. They can hydrolyze endo- or exo-β− 1,4-, endo-β− 1,3-, endo-β− 1,3–1,4- or endo-β− 1,3(4)- glycosidic bonds in glucans (Lafond et al. 2012; Lin et al. 2025). Among them, endo-β− 1,4-glucanase (EC 3.2.1.4), exo-glucanase/cellobiohydrolase (EC 3.2.1.91) and β-glucosidase (EC 3.2.1.21) are involved in the breakdown of glucan-like substrates, endo-β− 1,3-glucanases or laminarinases (EC 3.2.1.39) hydrolyze β− 1,3-glycosidic bonds in glucans, endo-β− 1,3–1,4-glucanase or lichenase (EC 3.2.1.73) cleaves the β− 1,4-linkages adjacent to β− 1,3-glycoside bonds in mixed linked glucan, and endo-β− 1,3(4)-glucanase (EC 3.2.1.6) randomly breaks β− 1,3 or β− 1,4 bonds in β-D-glucan (Jin et al. 2023). β-glucanase is present in 14 GH families (5, 6, 9, 10, 12, 26, 44, 45, 64, 81, 128, 157, 158, and 162). According to their mechanism of action, these enzymes are further classified into endo-glucanases (ECs 3.2.1.4/3.2.1.6/3.2.1.39/3.2.1.71/3.2.1.73/3.2.1.75/3.2.1.151), exo-glucanases (ECs 3.2.1.58/3.2.1.74/3.2.1.91), and glucosidases (EC 3.2.1.21). β-glucanases are of interest due to their applications in the feed, food, and textile industries (Caseiro et al. 2022; Chang et al. 2021).

Regarding strategies that can be used to search for and identify enzymes from different environments that can only be laboriously recovered by current culture methods, data-intensive metagenomic approaches of complex microbial communities have become a powerful tool (Adegboye et al. 2021; Batista-García et al. 2016; Madhavan et al. 2017). Since microorganisms exhibit a high degree of genomic and metabolic flexibility, which allows them to adapt to extreme environmental conditions, they represent a reservoir of proteins/enzymes capable of withstanding extreme conditions usually found in several industries (Badhai et al. 2015; Verma and Satyanarayana 2020). Consequently, many extreme environments have been explored to obtain extremophilic xylanases (Fredriksen et al. 2019; Li et al. 2019; Verma and Satyanarayana 2020). The termite gut is one of such environments, presenting pH, oxygen, and substrate gradients (Brune 2014; Liu et al. 2011) that are difficult to reproduce in vitro but are similar to those found in industrial environments. Furthermore, since the majority of microorganisms in termite guts are unculturable, the use of whole DNA metagenomic studies is a tool of choice for the screening of alkaline, halotolerant, and halophilic enzymes (Arora et al. 2022; Ben Guerrero et al. 2015; Romero Victorica et al. 2020; Vikram et al. 2021).

In a previous study conducted by our research group, we utilized a shotgun sequencing approach to analyze gut microbiome samples from two termite species, which led to the identification of several bacterial candidate genes encoding enzymes with lignocellulose-degrading potential (Romero Victorica et al. 2020). Based on those results, the objectives of the current study were as follows: (1) to perform an in silico scan of metagenomic contigs to identify and characterize potential xylanases; (2) to clone and recombinantly express two GH10 xylanase candidates (Xyl10 C and Xyl10E) for subsequent biochemical and in silico structural characterization; (3) to characterize the composition of pretreated *S. bicolor* bagasse (SBB); (4) to analyze the hydrolysis products of both commercial substrate and pretreated SBB; and (5) to evaluate the antioxidant potential of the resulting xylo-oligosaccharides (XOs).

## Materials and methods

### Preparation of pretreated *S. bicolor* bagasse

A variety of *S. bicolor* L. Moench bagasse (SBB), with low lignin content, known as brown midrib (BMR), was obtained from the network of cultivars and experimental lines of the Pergamino Experimental Station of the National Institute of Agricultural Technology (INTA), Buenos Aires, Argentina. The material was air-dried and milled to an average particle size of ~ 2 mm. A suspension was prepared according to Finore et al. (2016) with modifications. The suspension contained 50 g of SBB suspended in 1000 mL of 2 N KOH and was prepared by magnetic stirring at room temperature for 72 h. The suspension was centrifuged at 10,000 rpm at 4 °C for 1 h. The supernatant was precipitated with cold ethanol 96% 1:1 (v/v), stored at − 20 °C overnight and then centrifuged at 10,000 rpm at 4 °C for 1 h. The pellet was dissolved in hot water and dialyzed against distilled water for 2 days, by using dialysis membrane tubes (12,000–14,000 MW cut-off, Spectra/pore®, Sigma-Aldrich, St. Louis, MO, USA). Afterward, the material was lyophilized and the yield expressed as weight percentage with respect to the initial dry biomass (%, w/w) and used for the characterization of the amount and type of carbohydrates present. The material was also used as a substrate for the hydrolysis reactions.

### Chemical composition of pretreated SBB

The monomer composition of pretreated SBB was determined by chemical hydrolysis using 0.5 M trifluoroacetic acid (TFA). A polysaccharide sample (5 mg) was treated with 1 mL of TFA (Åman et al. 1981) and incubated at 120 °C for 2 h in a sealed tube. After cooling to room temperature, the sample was evaporated to dryness under a nitrogen atmosphere to remove completely the acid. Afterwards, 1 mL of methanol was added. The procedure was performed three times. This process facilitated the hydrolysis of the polysaccharides. The sample was resuspended in 500 µL of MilliQ water, and the resulting reaction solutions were centrifuged at 6000 rpm for 10 min. The hydrolysis products were analyzed by thin-layer chromatography (TLC, Silica GelF60, Merck Rahway, NJ) using *n*-BuOH/AcOH/H_2_O (6:2:2, by vol) as mobile phase and revealed with α-naphthol (Finore et al. 2016). The sugar composition was also identified by high-pressure anion exchange chromatography with a pulsed amperometric detector (HPAEC-PAD, Dionex ICS 5000 + DC, Thermo Fisher Scientific, Waltham, MA, USA) equipped with a CARBOPAC PA1 column, and the sugars were eluted isocratically with 16 mM NaOH; arabinose, galactose, glucose, and xylose were used as standards (Finore et al. 2016). The monomers were quantified using external calibration curves (Caruso et al. 2018). Total carbohydrate content was determined by the method of Dubois et al. (1956) using glucose as a standard. In addition, the ^1^H and ^13^C nuclear magnetic resonance (NMR) spectra of the hemicellulose fraction from pretreated SBB were determined on a Bruker 400 MHz Prodigy instrument (Bruker Scientific, Billerica, MA, USA) at 50 °C. For ^1^H analysis, the sample was exchanged three times with D_2_O with an intermediate freezing step at − 20 °C and lyophilizing step and then dissolved in 700 µL D_2_O. Chemical shifts are reported in parts per million, relative to D_2_O and CD_3_OD for ^1^H and ^13^C NMR spectra, respectively (Lo Giudice et al. 2020).

To complement the characterization of pretreated SBB, the gas chromatography–mass spectrometry (GC–MS) analytical technique was used according to Marques et al. (2010) with modifications. Approximately 5 mg of SBB was dissolved in 1 mL of 1.25 M anhydrous hydrochloric methanol. The methanolysis reaction was carried out at 80 °C for 16 h. The resulting methanolic phase was dried under a nitrogen flow and in a vacuum desiccator in the presence of CaCl_2_ for 20 min. Later, the sample was subjected to acetylation with 50 µL of pyridine and 50 µL of acetic anhydride at 100 °C for 30 min. Subsequently, the sample was dried under a nitrogen flow and then purified by performing extraction with chloroform/water (1:1 by vol) was carried out three times. The methanolic phase, containing the acetylated *O*-methyl glycosides, was dried, dissolved in 400 µL of acetone and then injected into the GC–MS using a Thermo Scientific Focus GC Series instrument (Thermo Fisher Scientific, Whaltman, MA, USA) equipped with a TG-SQC capillary column (Thermo Fisher Scientific, Whaltman, MA, USA), 30 m × 0.25 mm × 0.25 µm, flow 1 mL/min, He as carrier gas). The temperature program was 150 °C for 3 min, followed by a ramp of 3 °C/min until reaching 330 °C and not held.

### Bioinformatics analysis of the two new GH10 xylanases prospected from a termite gut microbiome

We have previously reported that the most abundant gene family involved in xylan degradation in the gut microbiome of *Nasutitermes aquilinus* is GH10 (Romero Victorica et al. 2020). From the assembled contigs, we selected two predicted GH10-encoding genes: KBCPBGKF 20803 and KBCPBGKF 45352 (hereafter termed Xyl10 C and Xyl10E, respectively). The criteria to select these two GH10-encoding genes were the following: (a) GH10 is the most abundant family involved in hemicellulose deconstruction identified in the gut microbiome of *N. aquilinus* analyzed by Romero Victorica et al. (2020), (b) the encoding genes present a complete coding sequence containing identifiable start and stop codons and a complete reading frame, and (c) the encoding genes have a signal peptide, which suggests that it could encode a secreted enzyme.

According to an initial BLASTP analysis against the non-redundant protein sequences database, the first 15 hits for each enzyme resulted to be the same accessions, except one, which was present only in Xyl10E, and were included as reference sequences. The 18 sequences were pooled and aligned with the Muscle algorithm as implemented in MEGA 11 (Edgar 2004; Tamura et al. 2021) with the default parameters. Subsequently, the resulting multiple alignment was used to calculate a Neighbor Joining phylogenetic tree with 1000 bootstrap replicates, using MEGA 11. In addition, the peptides were functionally characterized using the InterProScan software of InterPro (https://www.ebi.ac.uk/interpro/).

### Cloning, expression, and protein purification of the heterologous proteins

Total DNA from *N. aquilinus* gut samples was extracted by using the commercial DNA extraction kit QIAamp DNA Stool (Qiagen, Hilden, Germany) following the manufacturer’s indications with modifications. Briefly, pooled gut samples from ten individuals were heated at 95 °C in 1 mL of lysis buffer, followed by a bead-beating step using FastPrep®− 24 (MP Biomedicals Inc., Irvine, CA, USA) with three cycles of 20 s at 6000 rpm, using 300 mg of glass beads (150–212 µm beads) (Sigma-Aldrich, St. Louis, MO, USA). Afterward, an extra magnetic purification step was performed with 1.5 volumes of magnetic bead solution (AgencourtAMPure XP magnetic beads, Beckman Coulter, Brea, CA, USA) for 5 min, followed by two washes with 80% ethanol.

The Xyl10 C and Xyl10E sequences were amplified, without that for the native signal peptide, with specific primers containing the *Bam*HI and *Xho*I restriction enzyme sites (shown underlined) (Xyl10 C-F: 5′GGATCCGCCTGTAAAACAAAC3′, Xyl10 C-R: 5′CTCGAGTTATTTAACCAACTCCC3′ and Xyl10E-F:5′ GGATCCTTCTGCGCCTGACA 3′, Xyl10E-R: 5′ CTCGAGCTATTCCACCAATTTCC 3′) for N-terminal fusion of the encoded enzyme to 6xHis. The amplification products were purified and cloned in the pGEM-T Easy vector (Promega, Madison, WI, USA), using *Escherichia coli* DH5-α competent cells (Thermo Fisher Scientific, Waltham, MA, USA). Then, the plasmid inserts from positive colonies were subcloned into a pET28b (+) vector (Novagen, Birmingham, UK) previously digested with *Bam*HI and *Xho*I (Novagen, Birmingham, UK) and transformed into competent *E. coli* Rosetta (DE3) cells (Novagen, Birmingham, UK). Cells were induced with 1 mM isopropyl-β-D-thiogalactopyranoside for 4 h at 28 °C and 37 °C, respectively. Induced cells were centrifuged at 6000 rpm at 4 °C, for 20 min. The resulting pellet was resuspended in 10 mL lysis buffer “A” pH 8/100 mL initial culture, containing 50 mM NaH_2_PO_4_, 300 mM NaCl, 10 mM Imidazole, and 0.1% (v/v) protease inhibitor cocktail (Thermo Fisher Scientific, Waltham, MA, USA). Resuspended cells were subjected to enzymatic lysis (1 mg/mL lysozyme; Thermo Fisher Scientific, Waltham, MA, USA) for 30 min on ice. Also, sonication cell lysis was implemented, on ice (six pulses of 10 s, 28% amplitude) in a Branson Sonifier 250 (VWR Scientific, Atlanta, GA, USA) and subsequently centrifuged at 11,000 rpm at 4 °C, for 30 min. The supernatant was filtered with 0.22 µm cellulose membrane filters (Ministart, Sartorious Stedium Biotech, Boston, MA, USA) and transferred to clean tubes. An aliquot of Ni–NTA agarose resin (Qiagen, Hilden, Germany) was washed with lysis buffer “A”, three times, and bound with the Xyl10 C and Xyl10E enzymes at 4 °C for 16 h. Afterwards, the sample was transferred to an Econo-Pac gravity flow purification column (Bio-Rad, Irvine, CA, USA). The flow-through was passed through the column twice, and the enzymes bound to the resin were washed three times (4 mL) with 50 mM NaH_2_PO_4_, 300 mM NaCl, and 20 mM Imidazol as washing buffer and eluted with 50 mM NaH_2_PO_4_, 300 mM NaCl, and 250 mM imidazole, at pH 8, as elution buffer. Protein concentrations were estimated using Bradford Reagent (Bio-Rad, Irvine, CA, USA) with bovine serum albumin as standard (Bradford 1976). The purification process yielded 1.2 mg (Xyl10 C) and 1.5 mg (Xyl10E) of purified soluble active recombinant protein from 100 mL of induced recombinant *E. coli* cultures.

The purified proteins were boiled at 100 °C with sample buffer containing β-mercaptoethanol for 5 min, and electrophoretically separated on 12% sodium dodecyl sulfate polyacrylamide gel electrophoresis (SDS-PAGE) and visualized with Coomassie Brilliant Blue R- 250 staining. Also, a western blot with anti-His mouse antibody (GE Healthcare Life Science, Rahway, NJ, USA) and anti-mouse alkaline phosphatase conjugated goat antibody (Sigma-Aldrich, St. Louis, MO, USA) was performed to detect the recombinant proteins using the BCIP/NBT (Promega, Madison, WI, USA) substrate as developing system. A pre-stained PageRuler Plus protein ladder (10–250 kDa) (Thermo Fisher Scientific, Waltham, MA, USA) served as a molecular weight marker.

### Biochemical characterization of Xyl10 C and Xyl10E

The endoxylanase, β-glucanase, endomannanase, laminarase, exoglucanase, and endoglucanase activities of Xyl10 C and Xyl10E were assessed in 1% (w/v) of beechwood xylan, barley β-glucan, galactomannan, laminarin, Avicel, and carboxymethyl cellulose (CMC) (Sigma-Aldrich, St. Louis, MO, USA), respectively, in a final reaction volume of 0.1 mL, at 50 °C and 400 rpm, for 40 min in the case of Xyl10 C and 30 min in the case of Xyl10E, in a Thermomixer (Eppendorf, Hamburg, Germany). In addition, 1% (w/v) of pretreated SBB was used as substrate in a final reaction volume of 1 mL, at 400 rpm at 40 °C, for 120 min. The reducing sugars released from the hydrolysis of xylan, barley β-glucan, galactomannan, laminarin, Avicel, CMC, and pretreated SBB were determined using the 3,5-dinitrosalicylic acid assay at 540 nm with xylose or glucose as standards, respectively (Miller 1959). The reaction was modified to reduced volumes (Romano et al. 2013).

The arabinofuranosidase, cellobiohydrolase, β-glucosidase, and xylosidase activities of Xyl10 C and Xyl10E were evaluated using 4-nitrophenyl-α-L-arabinofuranoside (*p*NPA) (Megazyme, Bray, Ireland), 4-nitrophenyl-β-D-cellobioside (*p*NPC), 4-nitrophenyl-β-D-glucopyranoside (*p*NPG), and 4-nitrophenyl-β-D-xylopyranoside (*p*NPX) (Sigma-Aldrich, St. Louis, MO, USA). Reactions of 0.1 mL containing 2.5 mM of each substrate were prepared in 50 mM sodium phosphate buffer (pH 7) and the properly diluted enzyme solution. The mixtures were incubated at 50 °C for 20 min and, subsequently, the reaction was stopped by adding 0.5 mL of 2% Na_2_CO_3_. The concentration of the released *p*-nitrophenol (*p*NP) was determined by measuring the absorbance at 410 nm and referencing it to a standard curve. The enzyme activities are expressed as international units (IU)/mg of protein. One IU was defined as the quantity of enzyme releasing 1 µmol of xylose or glucose per minute under the specified conditions for all enzymatic assays, including reactions performed with pretreated SBB as substrate.

### Evaluation of Xyl10 C and Xyl10E xylanase activity at different conditions of pH and temperature

The optimal pH was assessed using sodium citrate (pH 3–6), sodium phosphate (pH 7–8) and glycine–NaOH (pH 9–10) buffers at 50 °C. The influence of temperature was evaluated by incubation at pH 7 (Xyl10 C) and pH 6 (Xyl10E) and at temperatures ranging between 20 and 70 °C.

The thermal stability was assessed by pre-incubating the enzymes at 40 °C, 50 °C, 60 °C, 70 °C, and 80 °C at different time intervals (0, 2, 4, 6, and 8 h). In addition, kinetic parameters were determined under optimal assay conditions using 0–50 mg/mL of beechwood xylan as substrate, 0–10 mg/mL of barley β-glucan, and the addition of 1 M, 3 M and 5 M NaCl (Xyl10 C) by fitting models to data with the GraphPad Prism software v 8.0 (http://www.graphpad.com/scientific-software/prism/).

### Effects of various modulators on the enzymatic activity of Xyl10 C and Xyl10E

The effects of various metal ions (CaCl_2_, CuSO_4_, NiCl_2_, MgCl_2_, MnSO_4_, and ZnSO_4_ at 10 mM) and chemical reagents (ethylenediaminetetraacetic acid (EDTA), SDS, Tween- 40, dimethyl sulfoxide (DMSO), β-mercaptoethanol at 0.5% and NaCl (1 M, 3 M, and 5 M)) on Xyl10 C and Xyl10E activities were determined by adding each of them in sodium phosphate and citrate buffers, respectively (pH 7.0 and 6.0) into the standard reaction and incubating at 50 °C for 40 min (Xyl10 C) and 30 min (Xyl10E). Beechwood xylan (1%), barley β-glucan (1%), and pretreated SBB were used as substrates. The activity observed in the absence of metal ions or reagents was considered 100% (control). Stability under salinity conditions was assessed by preincubating Xyl10 C at an appropriate dilution with NaCl (1 M, 3 M, and 5 M), at 40 °C, for 1 h, before the standard reaction. No addition of NaCl was used as control.

### Analysis of the hydrolysis reaction products of Xyl10 C and Xyl10E

The hydrolysis patterns obtained using commercial substrates, cello- oligosaccharides (COs), xylo-oligosaccharides (XOs) and pretreated SBB were qualitatively analyzed by TLC according to Finore et al. (2016). The substrates used were: beechwood xylan (1%), barley β-glucan (1%), cellobiose (C2), cellotriose (C3), cellotetraose (C4), cellopentaose (C5), cellohexaose (C6), xylobiose (X2), xylotriose (X3), xylotetraose (X4), and xylopentaose (X5) (10 mM; Megazyme, Bray, Ireland), and pretreated SBB (1%). Glucose (C1), xylose (X1) (10 mM; Sigma-Aldrich, St. Louis, MO, USA), COs, and XOs (C2-C5 and X2-X5) (10 mM; Megazyme, Bray, Ireland) were used as standards.

The TLC technique was carried out as described in the section “Chemical composition of pretreated SBB.” The enzymatic reactions were carried out separately with beechwood xylan (1% w/v) and barley β-glucan (1% w/v) in 100 µL of reaction mixtures over different times (30 min, 40 min, 1 h, 2 h, 3 h, and 16 h). In addition, the enzymatic reactions were performed with pretreated SBB (1% w/v) for 2 h and with COs and XOs (10 mM) for 1 h. The assays were conducted at 50 °C and pH 7 for Xyl10 C, pH 6 for Xyl10E, and pH 7 for Xyl10 C + Xyl10E.

The resulting sugars were also identified by HPAEC-PAD Dionex ICS 5000 + DC (Thermo Fisher Scientific, Waltham, MA, USA), equipped with a CARBOPAC PA1 column (Thermo Fisher Scientific, Waltham, MA, USA), the sugars were eluted isocratically with 16 mM and 333 mM NaOH. Arabinose, galactose, glucose, xylose, XOs, and COs were used as standards (Finore et al. 2016). The monomers were quantified using external calibration curves (Caruso et al. 2018). Time course degradation of Xyl10E using pretreated SBB was performed at 2, 4, 8, and 16 h. The yield of xylose and XOs was estimated by HPAEC-PAD, according to the following equations (Eqs. (1), (2)), respectively, as described by Zhang et al. (2022) and Sluiter et al. (2012), where xylose, refers to the xylose obtained from the hydrolysis reaction, xylan in SBB, refers to the presence of xylan in pretreated SBB, and total SBB refers to the amount of pretreated SBB used. On the other hand, XOs in enzymatic hydrolysis refers to any XO (X2-X5) obtained from enzymatic hydrolysis.

1 $$\text{Xylose }\left(\mathrm{\%}\right)=\frac{\text{Xylose }\left(\mathrm{g}\right)}{\text{Xylan in SBB }\left(\mathrm{\%}\right) \times \text{ total SBB }\left(\mathrm{g}\right) \times 1.13}\times 100\%$$

2 $$Y \left(\mathrm{\%}\right)=\frac{\text{XOS in enzymatic hydrolysis }\left(\mathrm{g}\right)}{\text{Xylan in SBB }\left(\mathrm{g}\right)}\times 100\mathrm{\%}$$

### Antioxidant activity of xylo-oligosaccharides

The enzymatic hydrolysis products obtained using Xyl10E on pretreated SBB were concentrated with a Vivaspin® 20 Ultrafiltration Centrifugal Concentrator (50 kDa cutoff, Ministart, Sartorius Stedim Biotech, Boston, MA, USA), followed by centrifugation at 3700 g for 60 min. The resulting concentrated sample was then lyophilized for further analysis. Since a 6-h hydrolysis time was found to be optimal for maximum XO production, the enzymatic hydrolysis was repeated multiple times to ensure an adequate quantity of XOs for further analysis.

The antioxidant activity of purified XOs was evaluated by the effect of scavenging 2,2-diphenyl- 1-picrylhydrazyl radicals (DPPH) according to Zhang et al. (2019). Briefly, an aliquot of 0.05 mL of purified XOs (3 and 1.5 mg/mL) was mixed with 1.45 mL of a 0.1 mM DPPH methanol solution. The mixture was shaken for 30 min in the dark and absorbance was measured at 515 nm. Radical scavenging activity (% inhibition) was calculated using Eq. (3), where Abs control is the absorbance of the reaction in the presence of methanol and Abs XOs sample is the absorbance of the reaction with XOs. An ethanol solution of Trolox (Sigma-Aldrich, St. Louis, MO, USA) was used as the positive control.

3 $${\% inhibition}=\frac{\text{Abs control}-\text{Abs XOs sample}}{\text{Abs control}} \times 100 \%$$

In addition, the 2,2′-azinobis-(3-ethylbenzothiazoline- 6-sulfonic acid) (ABTS• +) radical assay (Sigma-Aldrich, St. Louis, MO, USA) was performed using a 7-mM stock solution in 2.5 mM K_2_S_2_O_8_, which was maintained in the dark for 16 h. For the ABTS assay, a dilution was prepared with ethanol to achieve an absorbance of 0.7 at 734 nm. One milliliter of the ABTS reagent dilution was mixed with 0.01 µL of the sample. A Trolox solution (2.5 mM) (Sigma-Aldrich, St. Louis, MO, USA) was used as the positive control, while ethanol alone served as the negative control. The reaction was incubated at 30 °C for 6 min, after which the absorbance was measured at 734 nm. The percentage of inhibition (% inhibition) was calculated using Eq. (3). Appropriate dilutions of the purified XOs sample were evaluated.

### Molecular analysis of Xyl10 C and Xyl10E

The molecular models of the Xyl10 C and Xyl10E structures were developed with the Deep-learning-based Iterative Threading ASSEmbly Refinement (D-I-TASSER) method implemented in the web server (https://zhanggroup.org/D-I-TASSER/). D-I-TASSER is a new method extended from I-TASSER for high-accuracy predictions of protein structure and function predictions (Zheng et al. 2023). Docking models between ligands and enzymes were obtained by a molecular replacement method. Next, the ligand pose in this new docking context was optimized by the Energy Minimize application in MOE (v2020.0901; https://www.chemcomp.com), which calculates atomic coordinates that are local minima of a potential energy function MOE. The quality of the molecular structures was assessed at the SWISS-MODEL server (https://swissmodel.expasy.org/qmean/) by QMEAN Score (Benkert et al. 2011) and MolProbity Analysis (Chen et al. 2010). The structural alignment, active site analysis, ligand interaction, and image production were performed with the MOE and Chimera software (Pettersen et al. 2004). The enzyme interaction with reference ligands (xyloheptaose and cellohexaose) and a two-dimension interaction graph were further analyzed with the Ligplot^+^ software (Laskowski and Swindells 2011) and the active site pockets were further measured with the CASTp server (Tian et al. 2018).

### Statistical analysis

All the assays were carried out in triplicate. Two independent biological replicate assays were performed with equivalent results. The results are expressed as the mean ± one standard deviation of the triplicate measurements.

### Nucleotide and amino acid sequence accession numbers

The amino acid sequences of Xyl10 C and Xyl10E were deposited in the GenBank database with accession numbers WOYO7770 and WOYO7771, respectively. In addition, the nucleotide sequences of Xyl10 C and Xyl10E were deposited in the GenBank database as OR371491 and OR371492, respectively.

## Results

### Chemical composition of pretreated SBB

The sugar composition of pretreated SBB was analyzed through the hydrolysis of the polysaccharide in 0.5 M TFA and determined by TLC and HPAEC-PAD. This analysis revealed that pretreated SBB contained mainly xylose, arabinose, galactose, and glucose in a relative molar proportion of 1: 0.12: 0.072: 0.0013 (Supplemental Fig. S1a and b). The material extracted with 2 N KOH represented 15% (w/w) of the dried SBB. The carbohydrate content, determined by the Dubois method, revealed that the sample was composed of 0.84 ± 0.01 g/L carbohydrates.

**Fig. 1:**
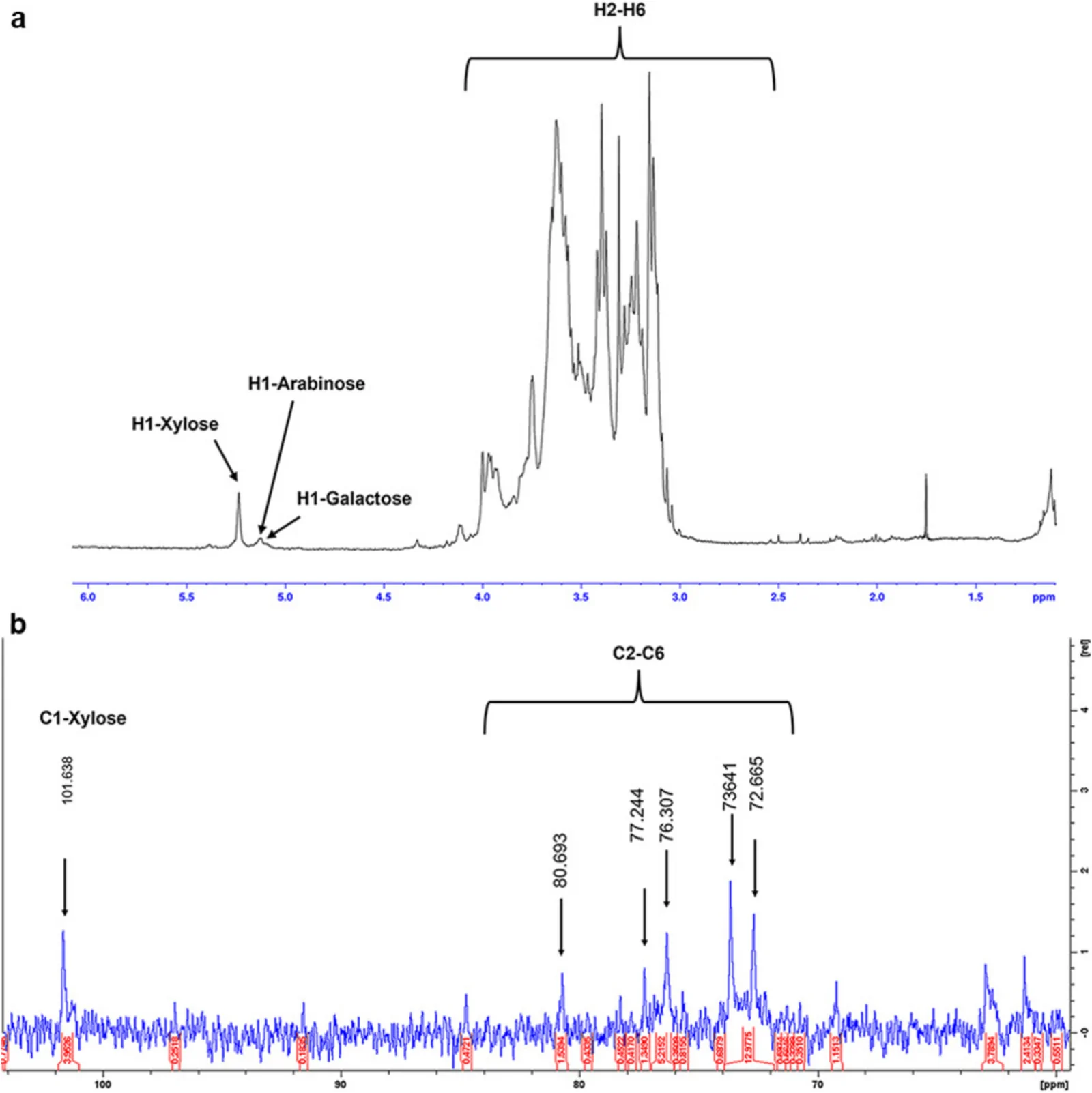
Nuclear magnetic resonance spectroscopy (NMR) analysis of polysaccharides recovered after SBB pretreatment; ^1^H (upper) (**a**) and ^13^C (lower) traces were recorded with a Bruker 400 MHz Prodigy instrument (**b**). The x-axis for each spectrum is expressed as chemical shifts reported as parts per million (ppm) with reference to D_2_O and CD_3_OD for the ^1^H and ^13^C spectra, respectively

In addition, the polysaccharide recovered after SBB pretreatment was further investigated using GC–MS and NMR techniques. Based on the analysis by mass spectra fragmentation, the polymer was composed of the following monomers: xylose (52.91%), arabinose (29.80%), glucose (14.16%), and galactose (3.11%) (Supplemental Fig. S2). The analysis of the ^1^H-NMR spectrum allowed identifying three main signals at δ 5.237 ppm, δ 5.130 ppm, and δ 5.122 ppm in the anomeric proton region, attributable to the three most abundant monomer residues, previously identified by hydrolysis, i.e., xylose, arabinose, and galactose, in an α-anomer configuration (Fig. 1a). The analysis of the ^13^C-NMR spectrum allowed clearly identifying, in the anomeric region, only the signal at δ 101.638 ppm of the most abundant residue, as indicated by chemical hydrolysis. Other signals were detected in the region from 90 to 60 ppm, attributable to the carbon ring resonances of the monomers identified in the polysaccharide structure, as also previously reported for other plant waste-derived polysaccharides (Fig. 1b) (Di Donato et al. 2020).

### Sequence and phylogeny analysis of Xyl10 C and Xyl10E

A BLASTP analysis of Xyl10 C against the non-redundant database of GenBank showed that the records with highest similarity were MDR2942514.1, an endo-β− 1,4- xylanase from *Treponema* sp. (85.19% identity, E value 0.0, and 100% coverage), and UKT59741.1 (Xyl10B), a GH10 xylanase found in a termite gut metagenome and previously characterized by our group (85.91% identity, E value 0.0, and 97% coverage) (Mon et al. 2022). A similar BLASTP search for Xyl10E revealed that the highest similarity corresponded to MDR0456598.1, an endo-β− 1,4-xylanase from *Treponema* sp. (70.60% identity, E value 0.0, and 86% coverage). Both MDR2942514 and MDR0456598 originated in the same survey of metagenome-assembled genomes from termite guts (GenBank: PRJNA732531) (Salgado et al. 2024).

A multiple sequence alignment of the amino acid sequences of Xy110 C, Xy110E, and a set of proteins belonging to the GH10 family showed a high level of conservation in the catalytic region of the GH10 family (Supplemental Fig. S3). The glutamic acid positions of the catalytic site are completely conserved. Also, the two positions responsible for the interaction mainly with XO and CO ligands are highly conserved, with a glutamine in both sites in all sequences but one. These glutamines were detected in a bifunctional GH10 reported by Xue et al. (2015). An InterProScan analysis of domains in the amino acid sequences of Xyl10 C and Xyl10E identified the presence of a GH10 domain motif (InterPro code IPR001000), spanning positions 15 to 378 and 72 to 434, respectively.

The phylogenetic analysis of Xyl10 C and Xyl10E and a group of similar GH10 reference sequences retrieved by BLASTP from the non-redundant protein sequences database revealed that Xyl10 C grouped with Xyl10B (UKT59741.1), the abovementioned GH10 endo- 1,4-β-xylanase, previously identified from the gut microbiome of *N. aquilinus* (Mon et al. 2022), and close to an endo-β− 1,4-xylanase from *Treponema* sp. (MDR2942514) identified in the termite gut microbiome. The analysis also revealed that Xyl10E grouped with an endo- 1,4-β-xylanase from *Treponema* sp. (MDR0456598) identified from metagenome-assembled genomes from termite guts (Supplemental Fig. S4).

### Cloning and heterologous expression of Xyl10 C and Xyl10E

**Fig. 2:**
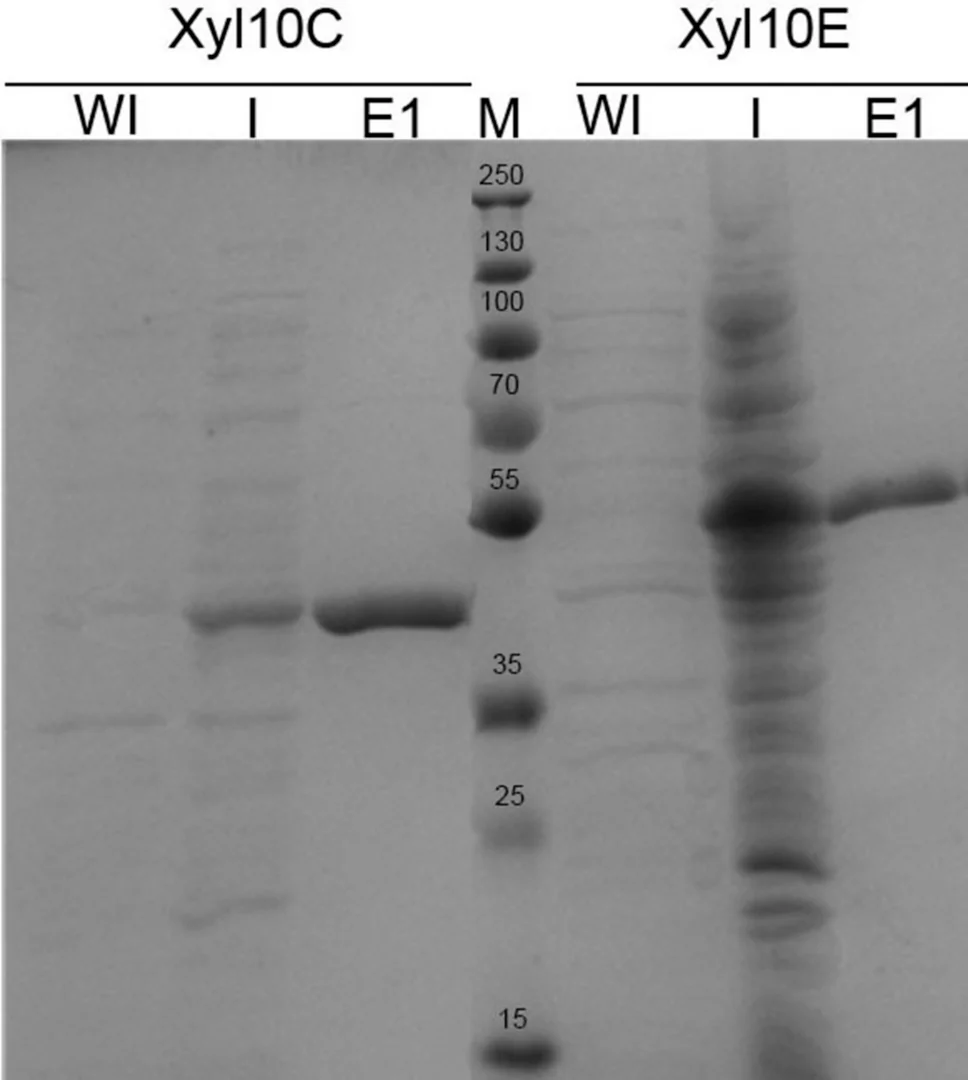
SDS-PAGE analysis of purified Xyl10 C and Xyl10E. Soluble immobilized metal-affinity chromatography (IMAC) purification (12% SDS-PAGE) stained with Coomassie blue. Prestained protein marker (M); total protein content of uninduced culture lysate (WI); total protein content of induced culture lysate (I); first purified elutions (E1) of each xylanase enzyme

The cloning and recombinant expression of Xyl10 C and Xyl10E expressed as a 6xHis N-terminal fusion proteins without signal peptide yielded recombinant proteins of 46.8 kDa and 49.2 kDa, respectively, i.e., very similar to the deduced molecular mass of both proteins, as estimated by SDS-PAGE (Fig. 2) and western blot analyses (Supplemental Fig. S5).

### Characterization of the activities of the recombinant Xyl10 C and Xyl10E enzymes

We have previously demonstrated that Xyll0E has β-xylanase activity on beechwood xylan (Romero Victorica et al. 2020). To further characterize the substrate specificity of the enzymes here studied, we evaluated their activity on several commercial substrates and pretreated SBB. The analysis of the hydrolytic activities of Xyl10 C and Xyl10E on various commercial substrates showed high activity on beechwood xylan of 197.5 ± 1.6 IU/mg and 288.1 ± 0.1 IU/mg, respectively. In addition, Xyl10E showed activity on barley β-glucan of 140.8 ± 2.8 IU/mg. Also, both enzymes displayed low levels of cellobiohydrolase activity on *p*NPC (6.7 ± 0.1 and 18.4 ± 0.9 IU/mg, respectively). Furthermore, the hydrolytic activity of both enzymes on pretreated SBB showed values of 31.3 ± 1.3 IU/mg and 43 ± 0.6 IU/mg, respectively. However, no activity was detected on substrates *p*NPA,* p*NPG, *p*NPX, CMC, Avicel, laminarin, or galactomannan (Table 1).

**Table 1:** Substrate specificity of purified Xyl10 C and Xyl10E

Substrate		Activity	Product measured		Xyl10 C (IU/mg)	Xyl10E (IU/mg)	
Avicel^a^	Exoglucanase		Total soluble sugars	ND			ND
Barley β- Glucan^a^	β-Glucanase		Total soluble sugars	ND			140.8 ± 2.8
Beechwood xylan^a^	Xylanase		Total soluble sugars	197.5 ± 1.6			288.1 ± 0.1
Carboxymethyl cellulose^a^	Endoglucanase		Total soluble sugars	ND			ND
Galactomannan^a^	Endomannanase		Total soluble sugars	ND			ND
Laminarin^a^	Laminarase		Total soluble sugars	ND			ND
*p*NP-Arabinofuranoside^b^	Arabinofuranosidase		*p*NP	ND			ND
*p*NP-Cellobioside^b^	Cellobiohydrolase		*p*NP	6.7 ± 0.1			18.4 ± 0.9
*p*NP-Glucopyranoside^b^	β-Glucosidase		*p*NP	ND			ND
*p*NP-Xylopyranoside^b^	Xylosidase		*p*NP	ND			ND
Pretreated sorghum bicolor bagasse BMR^+^ (SBB)^a^			Xylose	31.3 ± 1.3			43 ± 0.6
Cellobiose^b^			Glucose	ND			ND
Cellotriose^b^				ND			ND
Cellotetraose^b^				ND			ND
Cellopentaose^b^				ND			ND
Cellohexaose^b^				ND			ND
Xylobiose^b^			Xylose	ND			ND
Xylotriose^b^				ND			1.15 ± 0.07
Xylotetraose^b^				0.99 ± 0.04			3.88 ± 0.04
Xylopentaose^b^				0.91 ± 0.1			2.6 ± 0.07

*ND* not detected, *IU* international units (µmol of product/min of reaction)

^a^1%

^b^10 mM

**Fig. 3:**
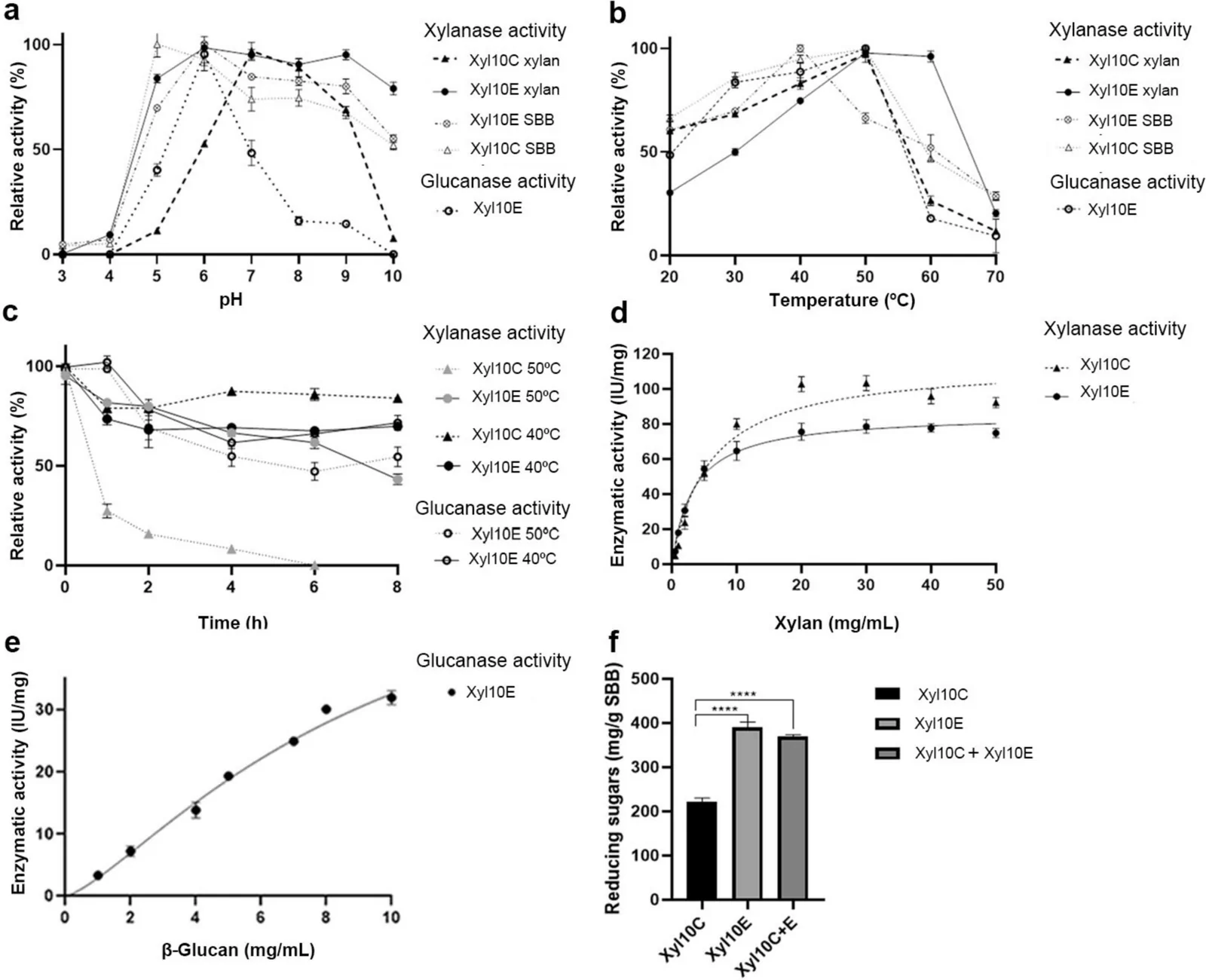
Enzymatic profile activity of Xyl10 C and Xyl10E. Optimal pH condition (**a**), temperature (**b**), thermal stability (**c**), and kinetic analysis (**d**, **e**), were evaluated using beechwood xylan, barley β-glucan and pretreated SBB. Concentration of reducing sugars released by hydrolysis of Xyl10 C, Xyl10E, and Xyl10 C + Xyl10E on pretreated SBB (2 h) (**f**). The results correspond to mean and standard deviations of technical triplicates. Two independent biological replicate assays were performed, with equivalent results. IU, international units: μmol/min

The analysis of the endo- 1,4-β-xylanase activity of Xyl10 C and Xyl10E on beechwood xylan at different pH and temperature values revealed that Xyl10 C had optimum activity at 50 °C and a pH range of 7.0–8.0 and that Xyl10E had optimum activity at a temperature range of 50–60 °C and a pH range of 5.0–9.0 (Fig. 3a and b). In addition, the endo- 1,4-β-xylanase activity of Xyl10E and Xyl10 C on pretreated SBB showed highest activity at 40 °C and 50 °C, respectively, and a pH range of 5.0–9.0. Furthermore, Xyl10 C and Xyl10E showed more than 85% and 70% of xylanase activity, respectively, after being incubated at 40 °C for 8 h (Fig. 3c). Besides, Xyl10E showed more than 70% β-glucanase activity at 40 °C preincubated for 8 h (Fig. 3c). Also, Xyl10E showed residual activity of 80% after 2 h of preincubation at 50 °C (Fig. 3c). The Xyl10 C and Xyl10E kinetic profiles on beechwood xylan at optimal pH and temperature were fitted to a Michaelis–Menten function (Fig. 3d). The *K*_M_, *V*_max_, and *K*_cat_ values of Xyl10 C were 5.72 mg/mL, 114.8 IU/mg, and 88.58 s^−1^, respectively. In addition, the catalytic efficiency (*K*_cat_/*K*_M_) was 15.48 mL/s·mg. For Xyl10E, the *K*_M_ was 3.106 mg/mL, the *V*_max_ was 82.83 IU/mg, and the *K*_cat_ was 67.11 s^−1^. Thus, the catalytic efficiency was 21.61 mL/s·mg (Fig. 3d). On the other hand, the kinetic profile of Xyl10E on barley β-glucan showed an almost sigmoidal profile (non-Michaelis Menten behavior), indicating positive cooperative binding (Fig. 3e). This finding suggests that Xyl10 C is a β-xylanase, with minor cellobiohydrolase activity, whereas Xyl10E is mainly a β-xylanase, which has glucanase activity to a lesser extent, with minimal cellobiohydrolase activity.

Furthermore, Xyl10 C kinetics on beechwood xylan under the presence of 3 M and 5 M NaCl exhibited *K*_cat_/*K*_M_ values 2.4- and 2.16-fold higher respectively than those of beechwood xylan with no addition of NaCl.

The hydrolysis of Xyl10 C or Xyl10E on pretreated SBB yielded 210 mg/g and 390 mg/g of reducing sugars from the substrate, respectively (Fig. 3f). Xyl10 C showed lower concentration of reducing sugars with respect to the combination of both enzymes with significant differences. In contrast, the use of Xyl10E alone showed no significant differences respect to the use of both enzymes simultaneously (Fig. 3f).

Regarding the effects of metal ions and chemicals on the xylanase activities of Xyl10 C and Xyl10E (Table 2), most of the metal ions and chemical agents used, such as Ca^2+^, Ni^2+^, Mg^2+^, and Zn^2+^, did not significantly reduce the activity of either enzyme, which maintained an activity greater than 80%; only Mn^2+^ strongly inhibited the activity of Xyl10E (30%) and Cu^2+^ maintained Xyl10E activity at a range of 70 to 75%. With respect to the other compounds evaluated, only SDS strongly inhibited the activities of both enzymes, while β-mercaptoethanol reduced the activity of Xyl10 C by ~ 45% (Table 2). In addition, Cu^2+^, Mn^2+^ and SDS significantly reduced the β-glucanase activity of Xyl10E. Regarding halotolerance, Xyl10 C was able to tolerate and even increase (~ 123% with NaCl 3 M) its activity at high salt concentrations with respect to the control (no added NaCl), while Xyl10E activity decreased at increasing NaCl concentrations (Table 2, Supplemental Fig. S6).

**Table 2:** Effect of metal ions and chemical reagents on the catalytic activity of Xyl10 C and Xyl10E

Chemical	Relative activity			
	Xyl10 C Xylanase	Xyl10E Xylanase	Xyl10E Glucanase	Xyl10E SBB
Control	98.99 ± 0.9	98.38 ± 2.3	98.17 ± 2.6	99.83 ± 1.9
β-Mercaptoethanol 0.5%	46.02 ± 0.4	83.52 ± 0.6	76.19 ± 15.3	86.52 ± 0.1
CaCl_2_ 10 mM	98.14 ± 1.6	95.52 ± 2.9	122.98 ± 2.0	95.28 ± 2.8
CuSO_4_ 10 mM	75.63 ± 2.7	70.22 ± 6.5	26.43 ± 2.1	97.66 ± 1.9
DMSO 0.5%	96.97 ± 3.4	97.37 ± 1.8	94.36 ± 6.6	93.47 ± 0.9
EDTA 0.5%	80.92 ± 2.7	82.68 ± 0.5	103.34 ± 2.2	51.50 ± 1.1
MgCl_2_ 10 mM	100.01 ± 5.0	77.39 ± 1.4	117.74 ± 4.7	83.07 ± 9.1
MnSO_4_ 10 mM	104.08 ± 5.5	31.26 ± 1.3	24.82 ± 2.2	61.71 ± 5.1
NaCl 1 M	114.90 ± 0.1	78.72 ± 2.3	78.05 ± 1.3	90.19 ± 1.8
NaCl 3 M	122.91 ± 0.4	48.53 ± 0.1	39.76 ± 1.7	65.85 ± 2.3
NaCl 5 M	114.33 ± 0.4	26.78 ± 0.8	41.01 ± 1.6	69.68 ± 2.5
NiCl_2_ 10 mM	86.02 ± 2.4	91.20 ± 4.5	95.29 ± 18.5	92.13 ± 5.9
SDS 0.5%	1.97 ± 1.2	7.93 ± 0.1	15.25 ± 3.8	10.04 ± 0.9
Tween 40 0.5%	95.16 ± 0.4	92.69 ± 0.5	124.71 ± 1.2	95.13 ± 9.2
ZnSO_4_ 10 mM	91.50 ± 4.2	95.06 ± 3.5	108.17 ± 3.9	98.82 ± 6.6

### Analysis of hydrolysis products

**Fig. 4:**
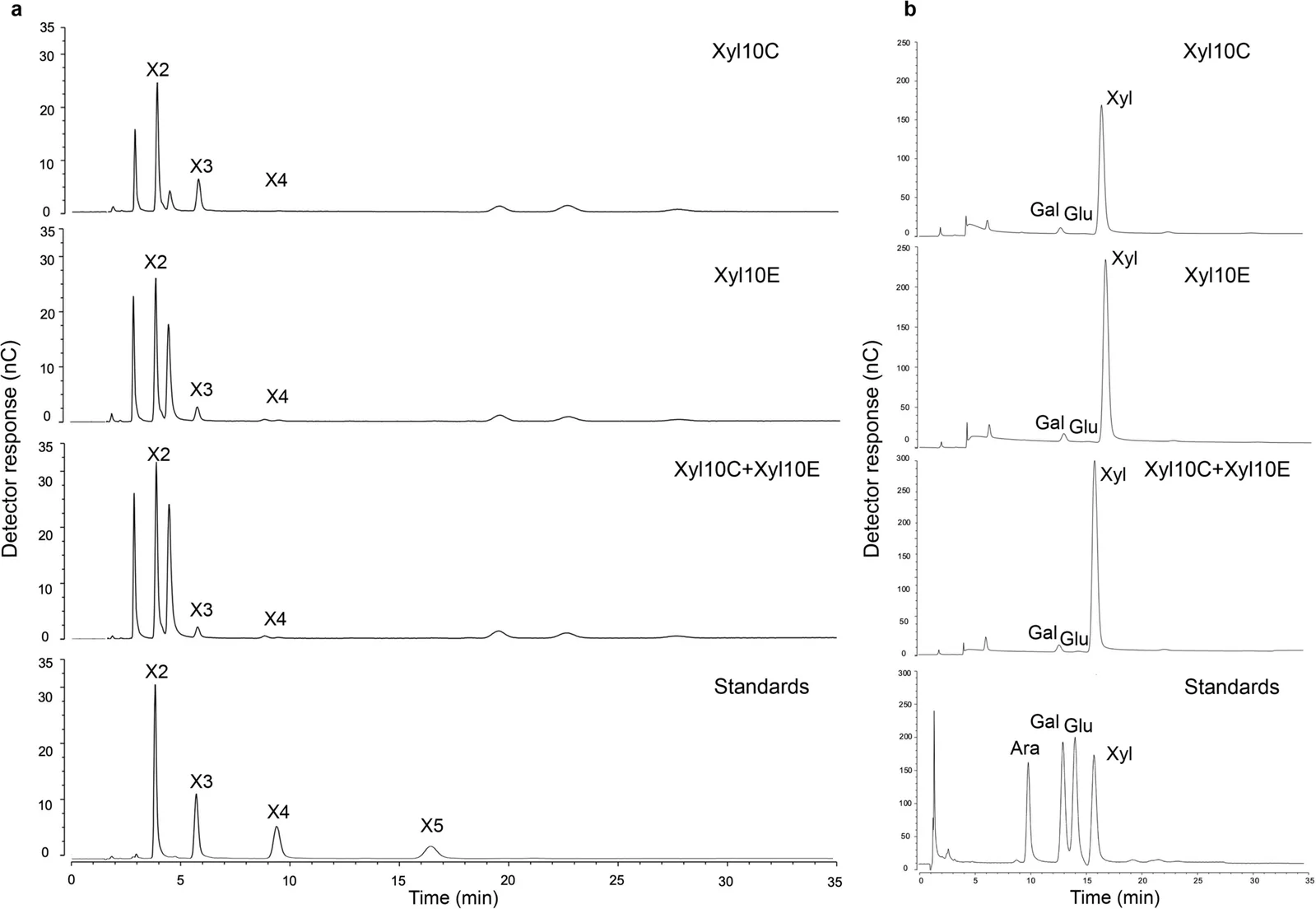
High-performance liquid chromatography (HPLC) hydrolysis profiles using Xyl10 C, Xyl10E, and their combination on pretreated SBB**.** Analysis of the xylo-oligosaccharides (XOs): xylobiose (X2), xylotriose (X3), xylotetraose (X4), and xylopentaose (X5) as standards, sample dilution 1:500 (**a**). Analysis of monosaccharides: arabinose (Ara), galactose (Gal), glucose (Glu), and xylose (X) as standards, sample dilution 1:10 (**b**)

The mode of action of Xyl10 C and Xyl10E was evaluated on beechwood xylan, COs, XOs, barley β-glucan, and pretreated SBB as substrates, and visualized in TLC and high-performance liquid chromatography (HPLC) (Fig. 4 and Supplemental S7). The activity profile of both enzymes on beechwood xylan revealed an evident increase in XOs content over time up to 16 h (Supplemental Fig. S7a and c). Thus, for both enzymes the degree of polymerization of XOs of > 6 xylose residues decreased during the hydrolysis, whereas the concentration of xylose (X1) and XOs of 2–4 xylose residues increased, being the proportion of X1 obtained with Xyl10E greater than that obtained with Xyl10 C (Supplemental Fig. S7a and c). When XOs were used as substrates, the TLC results showed that both enzymes hydrolyzed X4 releasing X1, X2, and X3 as products, that Xyl10 C hydrolyzed X5 to X2 and X3, and that Xyl0E hydrolyzed X5 to X1, X2, X3, and X4 (Supplemental Fig. S7b and d). Thus, both enzymes seem to present greater affinity for long-chain XOs. In the case of hydrolysis of barley β-glucan and COs with Xyl10E, the degree of polymerization of COs ≥ 3 increased over the course of hydrolysis when barley β-glucan was used as substrate (Supplemental Fig. S7e), but not when COs were used as substrate. In addition, the hydrolysis profiles of Xyl10 C and Xyl10E, both individually and in combination, using pretreated SBB as substrate, for a 2-h reaction, revealed that both the individual and their combination predominantly released X2, along with smaller quantities of X1, X3, X4, glucose, and galactose (Supplemental Fig. S7f, Fig. 4a, Table 3). The combined action of both enzymes led to an significant increase in the concentration of X2 compared to the individual enzyme Xyl10 C, with no significant difference observed when Xyl10E was used alone (Fig. 4a, Table 3). Considering this result and given that the use of Xyl10E alone showed no significant differences in reducing sugars concentration compared to the simultaneous use of both enzymes, the subsequent experiments were carried out using Xyl10E alone.

**Table 3:** Production of monosaccharides and xylo-oligosaccharides after enzymatic hydrolysis of pretreated SBB. Values expressed in g L^−1^

Monosaccharides/xylo-oligosaccharides	Xyl10 C	Xyl10E	Xyl10 C + Xyl10E
	2 h	2 h	2 h
Arabinose	0.00 ± 0.00	0.00 ± 0.00	3.68E^−03^ ± 0.06
Galactose	0.016 ± 0.08	0.014 ± 0.05	0.029 ± 0.08
Glucose	0.001 ± 0.09	0.001 ± 0–06	0.001 ± 0.04
Xylose	0.290 ± 0.19	0.383 ± 0.17	0.633 ± 0.20
X2	0.935 ± 0.15	1.106 ± 0.30	1.33 ± 0.22
X3	0.615 ± 0.29	0.296 ± 0.23	0.295 ± 0.18
X4	3.77E^−08^ ± 0.01	1.02E^−07^ ± 0.02	6.67E^−08^ ± 0–03
X5	0.00 ± 0.00	0.00 ± 0.00	0.00 ± 0.00

The time course degradation of pretreated SBB by Xyl10E, over a 2- to 16-h reaction revealed an increase in the concentrations of X1 and X2 and a decrease in X3 over time. Additionally, the concentration of X4 began to decrease after 6 h (Supplemental Fig. S8, Supplemental Table S1). The highest yields of X1 (~ 8%) and X2 (~ 18%) during the degradation of 1% pretreated SBB by Xyl10E were detected at 6 h (Supplemental Table S1). The quantitative estimation of total XOs ranged from 17 to 26% from 1% pretreated SBB after Xyl10E hydrolysis at 40 °C for 2 to 16 h (Supplemental Table S1). The highest peak of X2 release from Xyl10E was observed at 6 h, with a value of 1.480 ± 0.07 g L^−1^ (Supplemental Table S2).

### Antioxidant activity of XOs

The DPPH radical-scavenging activity of XO mixtures obtained by the Xyl10E enzymatic hydrolysis on pretreated SBB exhibited a concentration-dependent antioxidant effect after 6 h. At concentrations of 3 mg/mL and 1.5 mg/mL, the scavenging activities of the XOs were 30.25 ± 3.3 and 25.02 ± 2.1%, respectively. Similarly, the antioxidant activity measured by the ABTS•^+^ radical assay also showed a dose-dependent response, with scavenging activities of 48.58 ± 1.1% at 3 mg/mL and 30.50 ± 2.2% at 1.5 mg/mL. These results indicate that the antioxidant capacity of the XOs increases with concentration, as observed in both the DPPH and ABTS assays.

### Structural analysis of Xyl10 C and Xyl10E

**Fig. 5:**
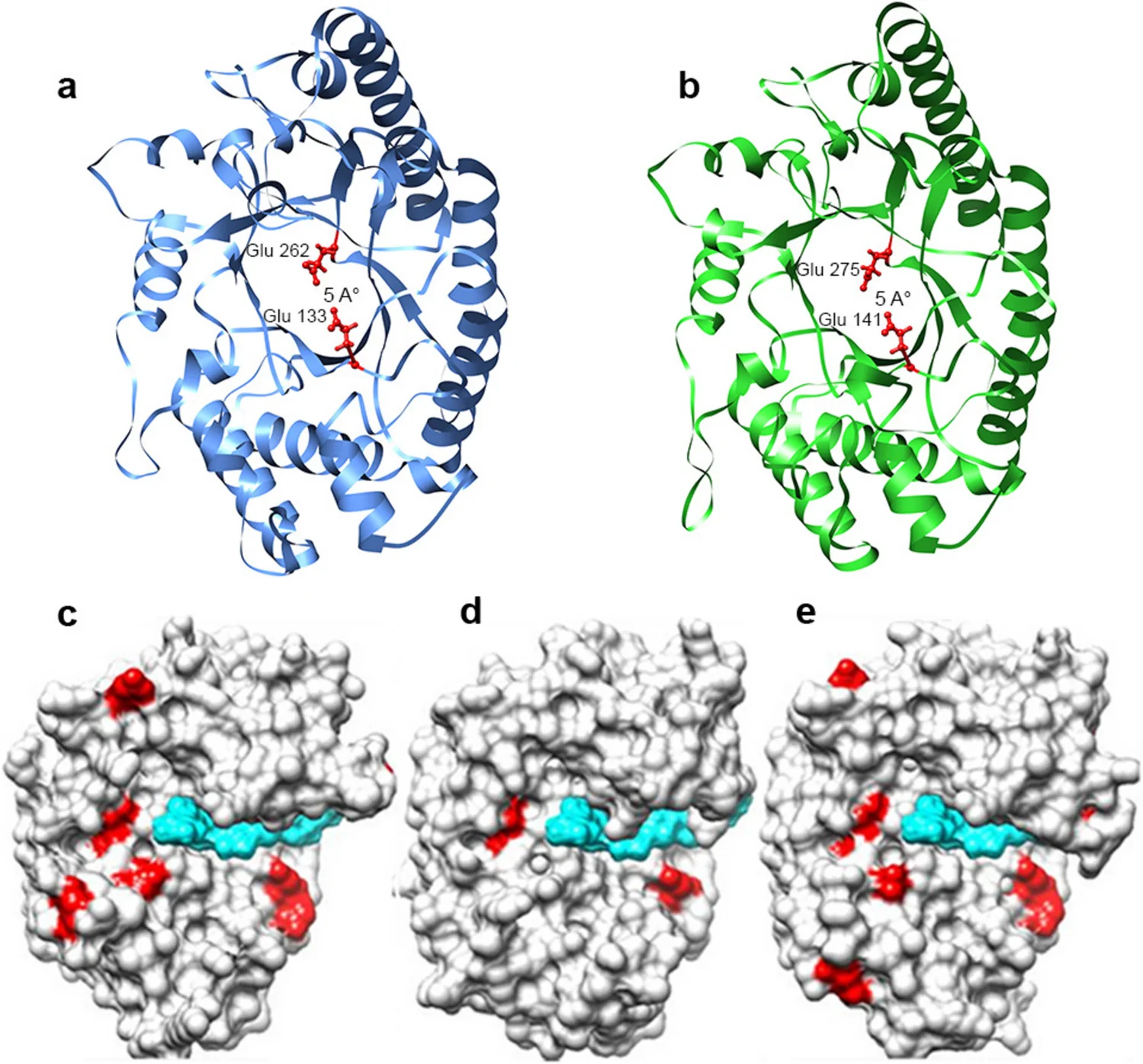
Modeled structure of Xyl10 C and Xyl10E. The complete TIM-barrel structures of Xyl10 C (**a**) and Xyl10E (**b**) with the catalytic glutamate residues are shown in red. Schematic representation of surface amino acids of Xyl10 C (**c**), Xyl10B (**d**), and Xyl10E (**e**). Red represents the acidic amino acids, whereas light blue represents the surface of the ligand in the catalytic site. Note the differential usage of the acidic amino acids that surround the catalytic site

The molecular models for the enzymes were developed by the D-I-TASSER and their quality estimated using diverse structure assessment tools. Specifically, the MolProbity score for Xyl10 C was of 1.05, whereas that for Xyl10E was of 1.23. The MolProbity score is adjusted to reflect the crystallographic resolution of the model, and a lower score means a better model (Chen et al. 2010). Then, the models obtained were of enough quality for the intended analysis. The results of the molecular structural analysis revealed that both proteins belong to the GH10 family and showed the representative (β/α)8 TIM barrel (triose-phosphate isomerase) fold of this family. Concerning the active site, both enzymes contain conserved residues as the canonical glutamate partners distanced by 5 Å. Specifically, the catalytic acid/base and the nucleophile residues identified were Glu 133 and Glu 262 for Xyl10 C and Glu 141 and Glu 275 for Xyl10E (Fig. 5a and b, respectively). Additionally, we measured the size of the catalytic pockets (as the area (Å^2^) and volume (Å^3^)) and found that the xylanase Xyl10E site was larger (1031, 1087) than that of Xyl10 C (873, 778). Moreover, we calculated the number of acid and basic residues and the resulting ratio (A/B ratio) as a descriptor associated with halotolerance, which is also illustrated as the higher number of acid residues counted for Xyl10 C compared to Xyl10E (non-halotolerant) and the halotolerant xylanase Xyl10B previously characterized by our group (Mon et al. 2022) (Fig. 5c and d). In this sense, the A/B value at the ligand binding face of the Xyl10 C was 3.3 (Supplemental Table S3).

The residues for the ligand binding site, specifically residues Glu 40, Asn 41, Lys 59, His 79, Trp 83, Gln 86, Asn 132, Glu 133, Tyr 208, Asn 209, Gln 240, Glu 262, Trp 322, Trp 330, and Ser 334 for Xyl10 C, and Glu 40, Asn 41, Lys 44, His 83, Trp 87, Gln 90, Asn 140, Glu 141, Tyr 206, Ser 207, Gln 238, Glu 275, Trp 329, Trp 337, and Arg 341 for Xyl10E, were also predicted by the D-I-TASSER. Based on the functional prediction, the top structural homologs for both enzymes were GH10 xylanases (PDB 1r87 A) and their ligand β-D-xylose.

**Fig. 6:**
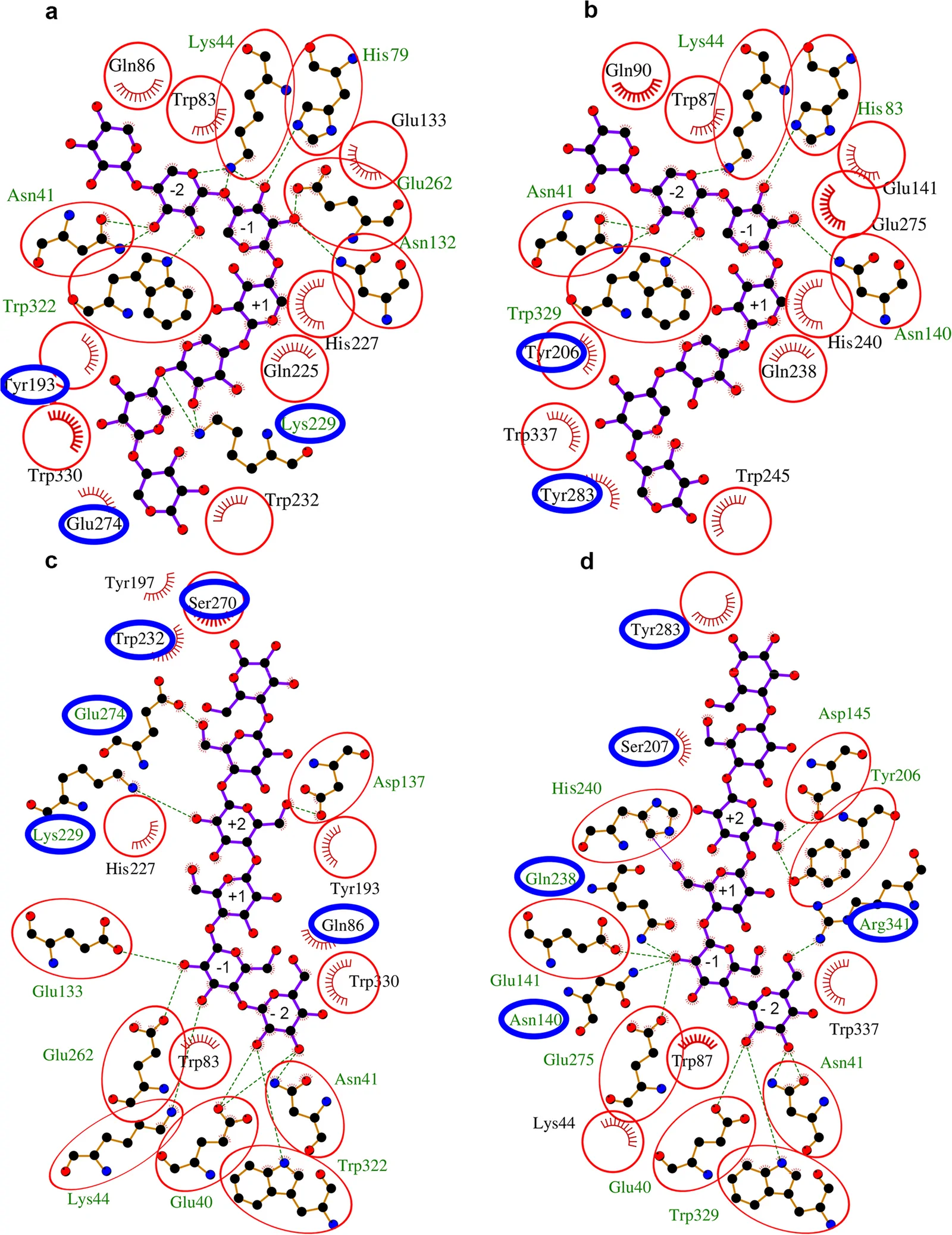
Molecular modeling of Xyl10 C and Xyl10E and their interaction with ligands. Schematic 2D Ligplot + representation of non-bonding interactions between the ligand xyloheptaose and Xyl10 C (**a**) and Xyl10E (**b**) and between the ligand cellohexaose and Xyl10 C (**c**) and Xyl10E (**d**). References in the Ligplot + are as follows: ligand residue names (blue), non-ligand residue names (olive green), H-bond labels (olive green), and hydrophobic residue names (black). References regarding atoms are as follows: nitrogen atoms (blue), oxygen atoms (red), and carbon atoms (black). References regarding bonds are as follows: ligand bonds external bonds (purple), non-ligand bonds (orange), hydrogen bonds (olive green), salt bridges (red), disulphide bonds (gold), external bonds (purple), and hydrophobic “bonds” (brick red). The red circles show the conserved residues in Xyl10 C and Xyl10E, whereas the blue circles indicate the differential amino acids

To gain a functional perspective of the enzymes, we next developed two docking models based on the structure of CbXyn10 C, a bifunctional enzyme of reference (Chu et al. 2017). Specifically, the interaction of CbXyn10 C with cellohexaose (PDB 5OFL) and xyloheptaose (PDB 5OFK) was set as a docking template where the ligand pose was docked by molecular replacement and relaxed at its new molecular pockets. Next, we implemented an analysis of the enzyme-ligand models with the LigPlot + software where the comparison of the interaction of both Xyl10 C and Xyl10E with the xyloheptaose ligand revealed that the interactions were similar. The differences were observed only in the interaction with Glu 274 and Tyr 193 in Xyl10 C and in the interaction with Tyr 283 and Tyr 206 in Xyl10E. Furthermore, Xyl10 C presented a Lys 229 which is absent in Xyl10E (blue circles, Fig. 6a, b, Supplemental Table S4).

However, the greatest number of differences was observed when cellohexaose was used as ligand. Xyl10E interacted with Tyr 283 and Arg 341, while Xyl10 C interacted with Ser 270 and Gln 86. Besides, Lys 229, Glu 274, and Trp 232 were present in Xyl10 C but not in Xyl10E (Fig. 6c and Supplemental Table S4). Thus, we observed a more divergent pattern of interaction for the enzymes with the cellohexaose ligand than for those with the xyloheptaose ligand (blue circles**,** Fig. 6a–d, Supplemental Table S4). Among the amino acids observed, His 240 and Gln 242 are involved in hydrogen bond interactions with cellohexaose but not in interactions with xyloheptaose in Xyl10E, some of which have already been observed by Chu et al. (2017), as important for the interaction with the secondary substrate.

## Discussion

In this study, we characterized the composition of pretreated SBB and the xylanase and β-glucanase activity of the recombinant enzymes Xyl10 C, Xyl10E, and their combination on both commercial substrates and pretreated SBB. We also analyzed hydrolysis products, assessed the antioxidant potential of XOs, and the structure of both enzymes.

SBB is considered an alternative for producing bioenergy and various bioproducts (Bonin et al. 2016). For this work, we used SBB, derived from a sorghum bagasse variety (BMR) due to its lower lignin content, and applied an alkaline pretreatment to enhance enzymatic accessibility. The pretreated SBB was primarily composed of xylose, with traces of arabinose, galactose, and glucose, as confirmed by NMR and GC–MS analyses. This suggests that the hemicellulose extract obtained was essentially present in a linear form. These results align with similar findings for NaOH and KOH pretreatment of SBB (Wei et al. 2018). Xyl10 C and Xyl10E, isolated from the microbiome of a termite gut, showed high identity with endo-β− 1,4-xylanases from *Treponema* sp. In the phylogenetic tree, both enzymes clustered with xylanases derived from termite gut microbiomes (Salgado et al. 2024), supporting their evolutionary adaptation to complex lignocellulosic substrates.

Xyl10 C demonstrated high substrate specificity for xylan, while Xyl10E exhibited bifunctionality, also acting on barley β-glucan. Notably, this is the first report of a GH10 bifunctional xylanase/β-glucanase from termite gut microbiomes.

Despite the high amino acid homology between Xyl10 C and Xyl10E, their enzymatic profiles are different. The most prominent difference is the bifunctionality of Xyl10E, a feature already described for other xylanases (Chu et al. 2017; Xie et al. 2021; Xue et al. 2015). From a mechanistic perspective, the bifunctionality is not related to major structural changes or multiple mutations, but rather to the sum of subtle differences (Andrews et al. 2000; Mendonça et al. 2023). In silico structural analysis of Xyl10E displayed a wider catalytic pocket, a feature associated with polyspecificity. Moreover, the average pocket of GH10 enzymes is wider and more functionally diverse than the narrow and more specific pocket of GH11 enzymes (Mendonça et al. 2023). The amino acid usage at the catalytic pocket has also been reported as a key element, where some amino acid substitutions that enhance flexibility have been associated with bifunctional properties. Specifically, Xyl10E displayed a Gln residue at position 46, in contrast to the Asp 46 residue of Xyl10 C, a mutation that has been proved to be beneficial for glucanase activity of a xylanase (Chu et al. 2017). Other differential residues that might account for flexibility near the catalytic niche are Ser 89 in Xyl10E vs Asn 85 in Xyl10 C. Differences that account for positive stacking interaction at the boundaries of the pocket are Tyr 283 vs Ser 270 in Xyl10E and Xyl10 C, respectively (Fig. 6c and d).

The biochemical characterization of Xyl10 C and Xyl0E on beechwood xylan revealed specific activities of approximately 198 IU/mg and ~ 288 IU/mg, respectively. These values are higher than those reported for other GH10 xylanases on commercial substrates, including rGH10XynA (~ 100 IU/mg), HC1 (~ 13 IU/mg) from *Paenibacillus* sp. (Ghio et al. 2018; Lee and Lee 2014), HY- 21 (~ 58 IU/mg) from *Cohnella laevirobosi* (Kim et al. 2010), XynRBM26 (~ 20 IU/mg) from *Massilia* sp. (Xu et al. 2016), and Pm25 (~ 7.4 IU/mg) from the gut microbiome of the termite *Pseudacanthotermes militaris* (Wu et al. 2021), and are similar in magnitude to those of Xyl10B (255 IU/mg) from the termite gut microbiota (*N. aquilinus*) (Mon et al. 2022), Xyn10 N18 (~ 242 IU/mg) from bovine rumen (Gong et al. 2013), and rXylR (~ 274.7 IU/mg) from *Duganella* sp. (Kim et al. 2021).

The optimal activity for Xyl10 C was observed at the neutral-alkaline pH range (7.0–8.0) and at temperature 40–50 °C, while Xyl10E exhibited a broader pH range (5.0–10.0) and temperature activity (50–60 °C), indicative of its enhanced versatility.

The kinetic profiles of Xyl10 C and Xyl10E on beechwood xylan were fitted to a Michaelis–Menten model, while Xyl10E showed an almost sigmoidal profile on barley β-glucan, suggesting a possible allosteric activation mechanism for substrate binding. This mechanism could be relevant for enzymes that cleave polymeric substrates, as it facilitates continuous substrate interaction after cleavage, like the behavior observed in other β-glucanases (Abel et al. 2003).

Both enzymes displayed high resistance to industrially relevant metal ions and chemical agents, maintaining over 80% activity in most conditions. Interestingly, Xyl10 C relative activity increased in the presence of NaCl, highlighting its halotolerance, which is supported by structural features such as a higher number of acidic amino acids in the catalytic pocket. Interestingly, the relative activity and the kcat/Km value of Xyl10 C increase presence of NaCl, compared to no added NaCl. In addition, a higher presence of acidic amino acids was observed in the catalytic region on the structural analysis*,* which is consistent with the halotolerant behavior. The amino acids acid could promote interactions with water molecules and ions, reducing repulsive electrostatic interactions (Graziano and Merlino 2014; Karan et al. 2020). This is the first report of a termite gut-derived xylanase with activity exceeding 100% under high-salinity conditions.

Xylanases and β-glucanases have diverse industrial applications. Xylanases are widely used as hemicellulose-degrading enzymes and are used in fruit juice processing, paper industry (Collins et al. 2005), second-generation ethanol production (Adegboye et al. 2021; Batista-García et al. 2016), XO production (de Freitas et al. 2019; Finore et al. 2016), and xylitol production (Venkateswar Rao et al. 2016). On the other hand, β-glucanases have potential application in the food, feed, and textile industries (Jin et al. 2023). Xylan- and β-glucan-degrading enzymes such as Xyl10E are usually used to decrease the viscosity of mash in the brewing industry since the raw materials (barley and malt) have considerable amounts of xylan and β− 1,3/1,4-glucan, which increase wort viscosity and decrease yields (Collins et al. 2005; Mon et al. 2022). Regarding Xyl10 C, its halotolerance makes it a promising enzyme for application in the seafood processing industries (Al-Darkazali et al. 2017; Verma and Satyanarayana 2020).

The enzymatic degradation products of both enzymes on beechwood xylan and pretreated SBB were characterized both by TLC and HPLC. Both Xyl10 C and Xyl10E released similar sugar profiles. When XOs were used as substrate, only larger XOs (X4 and X5) were hydrolyzed. Xyl10E also cleaved barley β-glucan, a linear glucan linked by β− 1,3 and β− 1,4 bonds, producing COs (≥ C3), consistent with previous studies (Abel et al. 2003). The main products of xylan degradation of Xyl10 C and Xyl10E on pretreated SBB was X2 followed by low amounts of X1, X3, and X4. Since the hydrolysates contained only small amounts of xylose, the xylanases characterized in this study can be classified mainly as endoxylanases, with minimal exoxylanase and/or β-xylosidase activity, although β-xylosidase activity was not determined. Previous studies have indicated that enzymes with high exoxylanase or xylosidase activities tend to produce substantial amounts of xylose, which may inhibit XO production (Bian et al. 2013; Vázquez et al. 2002).

Regarding pretreated SBB hydrolysis, Xyl10E released a higher amount of released reducing sugars compared to Xyl10 C. However, the combination of both enzymes did not significantly improve conversion efficiency. In addition, the X2 concentrations released from Xyl10E on pretreated SBB were higher than those from Xyl10 C and no significant difference was observed when both enzymes were used in combination, making Xyl10E the more sustainable choice for further applications.

Besides, since the concentration of reducing sugars showed no significant differences when using the combination of both enzymes on SBB with respect to the enzymes used individually, the time course degradation of SBB was assessed using Xyl10E. The highest peak of X2 release from Xyl10E was at 6 h (1.480 ± 0.07 g L^−1^) (Supplemental Table S2). These results are consistent with previous studies on sorghum biomass (Dos Santos et al. 2019), which reported X2 concentrations of 0.832 g L⁻^1^ after 12 h. In addition, the XOs production was in the same range (~ 26%) as that obtained in previous reports (~ 30%) obtained on sweet sorghum xylan (Joshi et al. 2020). Since the highest yields of X2 during the degradation of Xyl10E on pretreated SBB was detected at 6 h, the result of the hydrolysis reaction of Xyl10E on 1% pretreated SBB and 40 °C, at 6 h will be selected for further application using X2 and XOs.

XOs may be involved in several biological functions, including anti-inflammatory, antioxidant, antitumor, and antimicrobial properties (Abdella et al. 2021; Chen et al. 2021; Huang et al. 2022). X2 has been described to have the highest prebiotic activity in *Bifidobacterium* sp. proliferation and to be the XOs most suitable for food industry applications (Bhardwaj et al. 2019; Thirametoakkhara et al. 2023). Furthermore, since XOs are not decomposed by bacteria in the oral cavity, their use decreases the production of acidic substances and tooth decay (Samanta et al. 2015). Given the increasing global demand for antioxidants, it is essential to develop a cost-effective method to produce XOs from xylan-rich biomasses, including SBB. In this work, the antioxidant activity of XOs derived from pretreated SBB, assessed by DPPH and ABTS assays, was comparable to that of XOs obtained from other agricultural or agroindustrial residues, such as wheat husk and sugarcane bagasse (Bian et al. 2013; Jagtap et al. 2017).

Previous studies have investigated the production of XOs from SBB using fungal xylanase extracts, such as those from *Thermomyces lanuginosus* and *Aspergillus fumigatus*, which produced XOs with potential prebiotic effects (Nascimento et al. 2022; Ravichandra et al. 2023). Although a recombinant GH10 xylanase derived from a hot spring metagenome has been studied on sweet sorghum bagasse (Joshi et al. 2020), until now there have been no reports on the use of other recombinant GH10 xylanases to produce XOs from pretreated SBB. Furthermore, several studies have focused on using either crude or purified fungal xylanase extracts to produce XOs from various agro-industrial residues (Bagewadi et al. 2016; Dos Santos et al. 2019). Consequently, the production of XOs from pretreated SBB, particularly from the low lignin-containing BMR variety from Argentina, presents considerable biotechnological potential for a wide range of applications.

Concerning the molecular structure, both enzymes displayed a (β/α)8-barrel structural fold, which is a canonical structure for many glycosyl hydrolases, plus the catalytic mechanism, where two glutamic acid residues function as the proton donor and nucleophile, a fact shared by enzymes from the GH5 and GH10 families (Gebler et al. 1992; Jenkins et al. 1995; Mendonça et al. 2023). Interestingly, GH10 is a functionally diverse family and the referred catalysis between glutamic residues is a feature that supports such diversity (Glasgow et al. 2019).

In general, both enzymes share several common residues when interacting with the xyloheptaose ligand. Thus, this interaction is more conserved than the interaction of the two enzymes with the cellulose ligand (which is more divergent). Specifically, the interaction of Xyl10E with the cellohexaose ligand presents its catalytic Glu (141 and 275) surrounded by residues Gln 238 and Asn 140, which, due to their physicochemical properties, could favor the pKa of reactive glutamates improving the catalytic activity with this ligand.

In summary, this study demonstrates the biotechnological relevance of Xyl10 C and Xyl10E for SBB valorization, emphasizing Xyl10E bifunctionality, its efficiency in XO production, and the promising antioxidant activity of XOs derived from pretreated SBB, positioning them as potential candidates for biotechnological applications. The findings position these enzymes as potential candidates for biotechnological applications and provide a foundation for further exploration of termite gut-derived enzymes in sustainable industrial applications.

## Supplementary Information

Below is the link to the electronic supplementary material.

Supplementary file1 (DOCX 9466 kb)

## Data Availability

No datasets were generated or analysed during the current study.
