# Supplementary material for: Characterization of two GH10 enzymes with ability to hydrolyze pretreated Sorghum bicolor bagasse
Source: Appl Microbiol Biotechnol. 2025 Apr 28;109(1):104. doi: 10.1007/s00253-025-13484-4 (PMC12037437; doi:10.1007/s00253-025-13484-4)
Supplement: Supplementary file 1 — Supplementary file1 (DOCX 9466 kb) [file 253_2025_13484_MOESM1_ESM.docx]

**Supplemental Material**

**Characterization of two GH10 enzymes with ability to hydrolyze pretreated *Sorghum bicolor* bagasse**

Camila Bruno Baron^1,2^, María Laura Mon^1^, Rubén Marrero Díaz de Villegas^1^, Andrea Cattaneo^3^, Paola Di Donato^3,4^, Annarita Poli^3^, Maria Emilia Negri^5^, Mariana Alegre^5,6^, Marcelo A. Soria^7^, María Cecilia Rojo^8,9^, Mariana Combina^8,9^, Ilaria Finore^3^* and Paola M. Talia^1,2^*

^1^Instituto de Agrobiotecnología y Biología Molecular (IABIMO), UEDD INTA-CONICET, Hurlingham, Buenos Aires, Argentina

^2^Departamento de Biodiversidad y Biología Experimental, Facultad de Ciencias Exactas y Naturales, Universidad de Buenos Aires, Ciudad Autónoma de Buenos Aires, Argentina.

^3^Institute of Biomolecular Chemistry (ICB), Consiglio Nazionale delle Ricerche (CNR), Pozzuoli, Italy.

^4^Department of Science and Technology, University of Naples “Parthenope”, Napoli, Italy.

^5^Estación Experimental Agropecuaria Pergamino, Instituto Nacional de Tecnología Agropecuaria (INTA), Pergamino, Buenos Aires, Argentina.

^6^Escuela de Ciencias Agrarias y Ambientales-Universidad Nacional del Noroeste de la Provincia de Buenos Aires, Pergamino, Buenos Aires, Argentina.

^7^Cátedra de Microbiología Agrícola, Facultad de Agronomía, Universidad de Buenos Aires, INBA UBA-CONICET, Ciudad Autónoma de Buenos Aires, Argentina.

^8^Estación Experimental Agropecuaria Mendoza, Instituto Nacional de Tecnología Agropecuaria (INTA), Luján de Cuyo, Mendoza, Argentina.

^9^Consejo Nacional de Investigaciones Científicas y Tecnológicas (CONICET), Ciudad Autónoma de Buenos Aires, Argentina.

**ORCID:**

Camila Bruno Baron: 0009-0004-9273-514X

Maria Laura Mon: 0000-0003-1171-9013

Rubén Marrero Díaz de Villegas: [0000-0001-5250-6933](https://orcid.org/0000-0001-5250-6933)

Andrea Cattaneo: [0000-0002-1914-726X](https://orcid.org/0000-0002-1914-726X)

Paola Di Donato: 0000-0002-7485-1815

Annarita Poli: [0000-0001-6465-4765](https://orcid.org/0000-0001-6465-4765)

Maria Emilia Negri: 0009-0005-3851-6461

Mariana Alegre: 0009-0007-4283-5494

Marcelo A. Soria: 0000-0001-8556-147X

María Cecilia Rojo: 0000-0002-2834-8549

Mariana Combina: 0000-0002-0798-1564)

Ilaria Finore: 0000-0003-3606-5140

Paola M. Talia: [0000-0003-2877-8271](https://www.scopus.com/redirect.uri?url=http://www.orcid.org/0000-0003-2877-8271&authorId=16025322200&origin=AuthorProfile&orcId=0000-0003-2877-8271&category=orcidLink)

*Corresponding author. Tel: +54 11 46211447 Int 3627 / +39 081 867 5118

*E-mail address:* [talia.paola@inta.gob.ar](mailto:talia.paola@inta.gob.ar) / [taliapaolam@gmail.com](mailto:taliapaolam@gmail.com) / ilaria.finore@icb.cnr.it


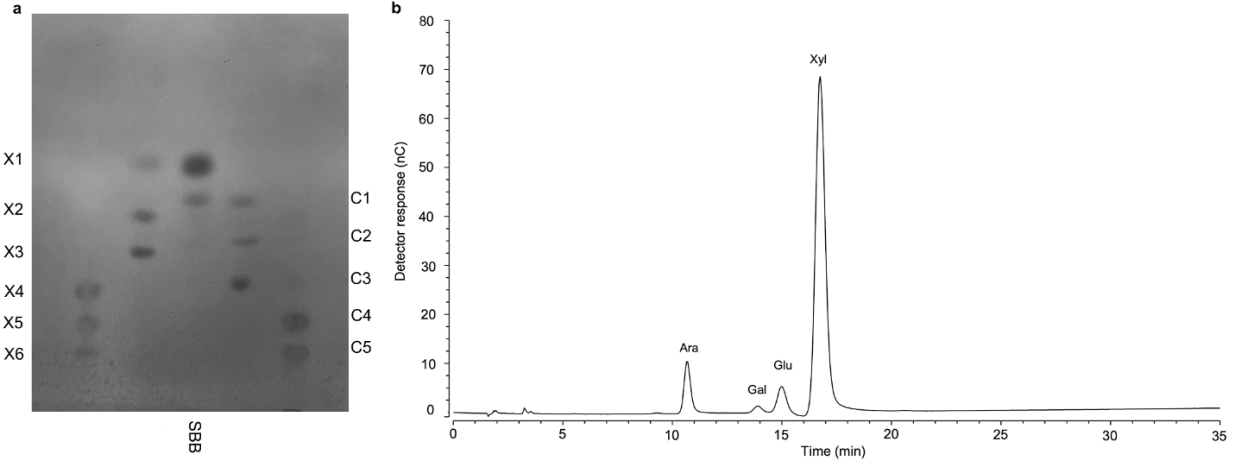


**Fig. S1 Characterization of pretreated SBB by hydrolysis with 0.5 M trifluoroacetic acid (TFA).** TLC of pretreated SBB using xylose (X1), xylobiose (X2), xylotriose (X3), xylotetraose (X4), xylopentaose (X5), xylohexaose (X6), glucose (C1), cellobiose (C2), cellotriose (C3), cellotetraose (C4) and cellopentaose (C5) as standards (a). HPLC chromatogram using arabinose (Ara), galactose (Gal), glucose (Glu) and xylose (Xyl) as standards (b).


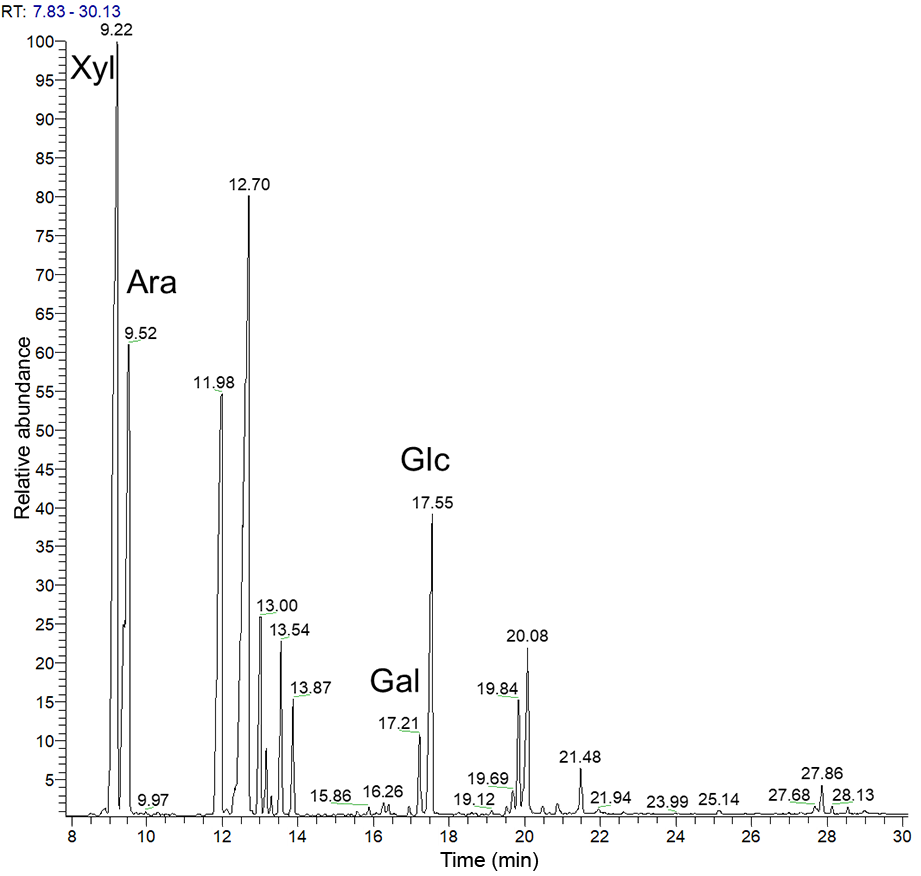


**Fig. S2 GC-MS chromatogram peaks of pretreated SBB.** The monosaccharides glucose (Glc), galactose (Gal), arabinose (Ara) and xylose (Xyl) were used as standards.


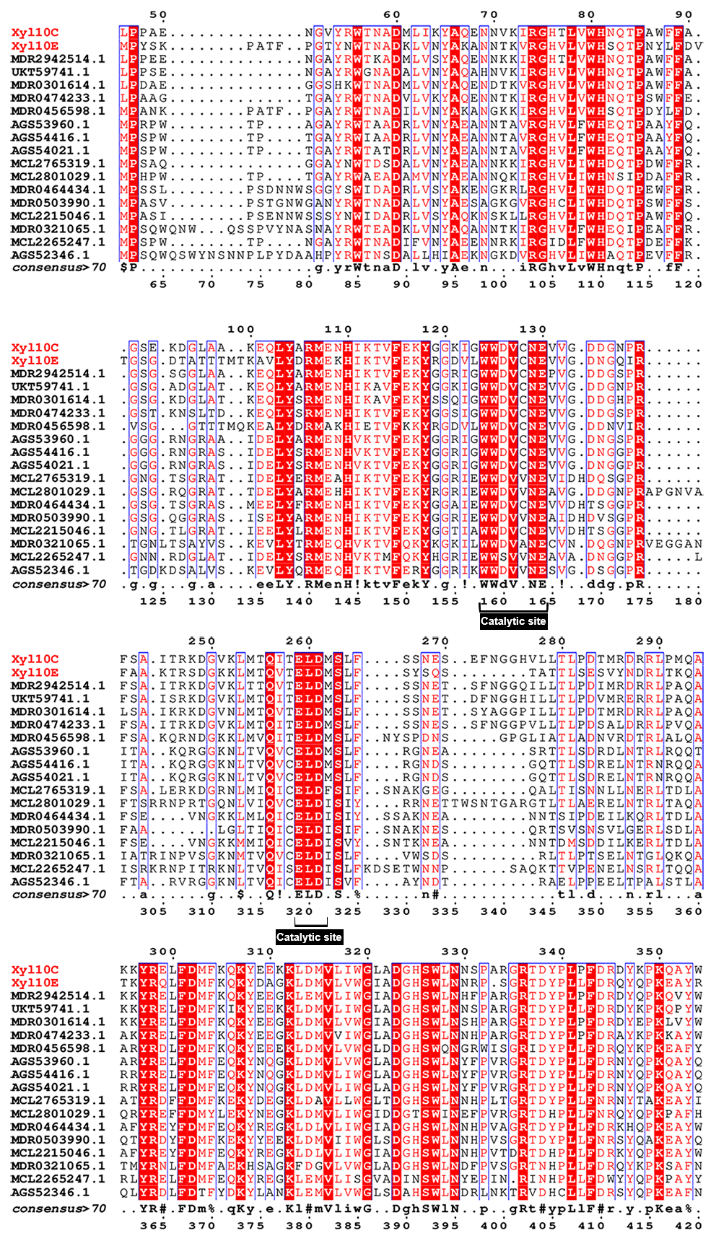


**Fig. S3 Amino acid sequence alignment of Xyl10C and Xyl10E with other GH10 enzymes**. The amino acid sequences were: UKT59741.1 from an uncultured bacterium (GenBank accession OK617332.1); AGS53960.1 from an uncultured bacterium (GenBank accession JQ844263.1); AGS54416.1 from an uncultured bacterium (GenBank accession JQ844297.1); AGS54021.1 from an uncultured bacterium (GenBank accession JQ844270.1); MCL2765319.1 from *Treponema* sp. termite gut metagenome (GenBank accession WQXU01000018.1); MCL2801029.1 from *Treponema* sp. termite gut metagenome (GenBank accession WQXI01000010.1); MCL2215046.1 from *Treponema* sp. termite gut metagenome (GenBank accession WQSQ01000054.1); MCL2184593.1 from *Treponema* sp. termite gut metagenome (GenBank accession WQRW01000006.1); MCL2265247.1 from *Treponema* sp. termite gut metagenome (GenBank accession WQTG01000021.1); AGS52346.1 from an uncultured bacterium (GenBank accession JQ844189.1); MCL2444490.1 from *Treponema* sp. termite gut metagenome (GenBank accession WQWT01000335.1); WP 015712816.1 from *Leadbettera azotonutricia* (GenBank accession NC015577.1); MCL2231419.1 from *Treponema* sp. termite gut metagenome (GenBank accession WQSL01000198.1); GHV71493.1 from a spirochete bacterium (GenBank accession BNUV01000074.1); and MCL2805240.1 from *Treponema* sp. termite gut metagenome (GenBank accession WQXG01000016.1). The black boxes indicate the conserved catalytic site, which contains the catalytic glutamic acid residues.


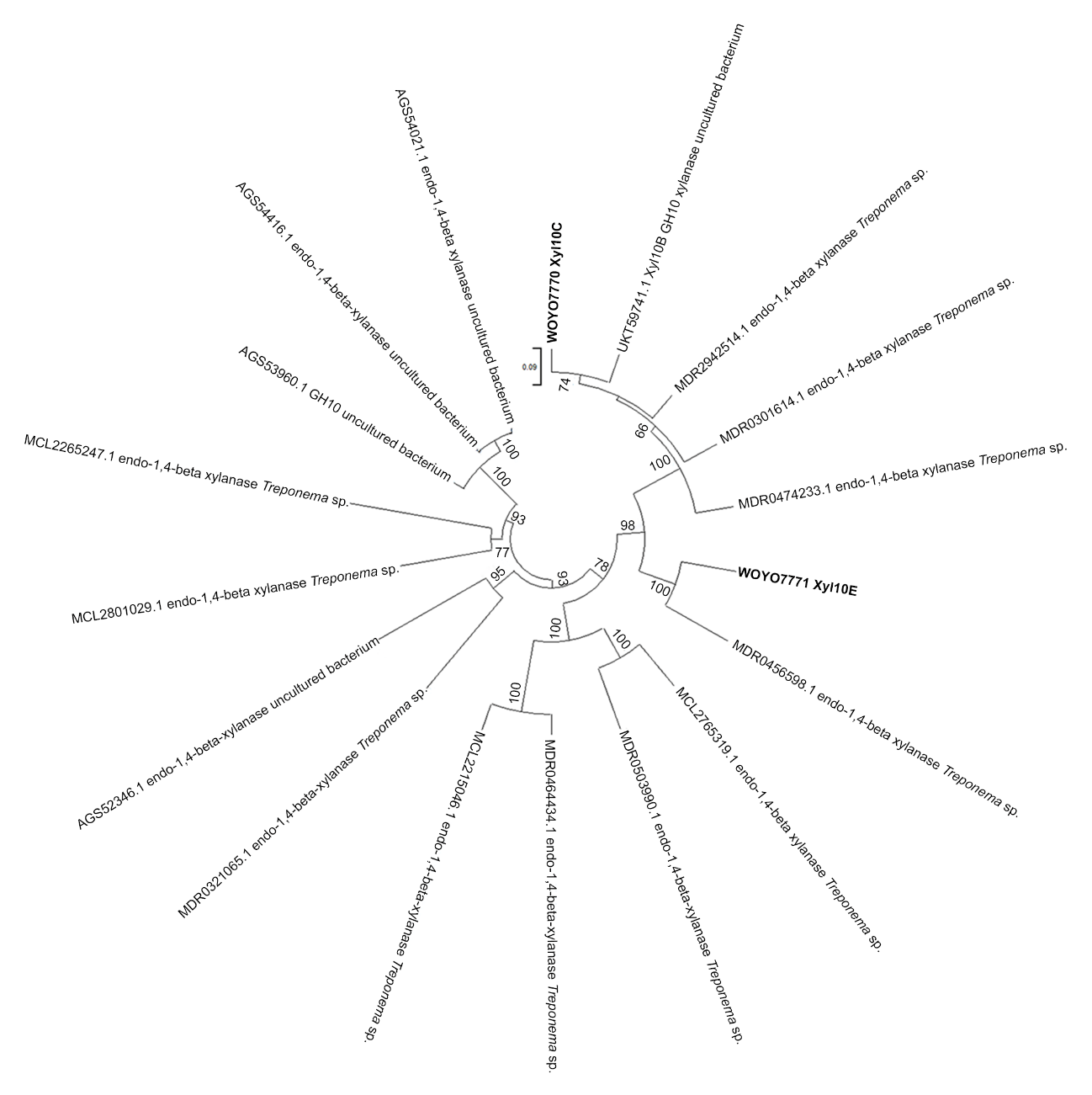


**Fig. S4 Phylogenetic relationships from Xyl10C and Xyl10E and reference sequences of glycosyl hydrolases family 10 inferred by the Neighbor Joining method.** Xyl10C and Xyl10E sequences are shown in bold.


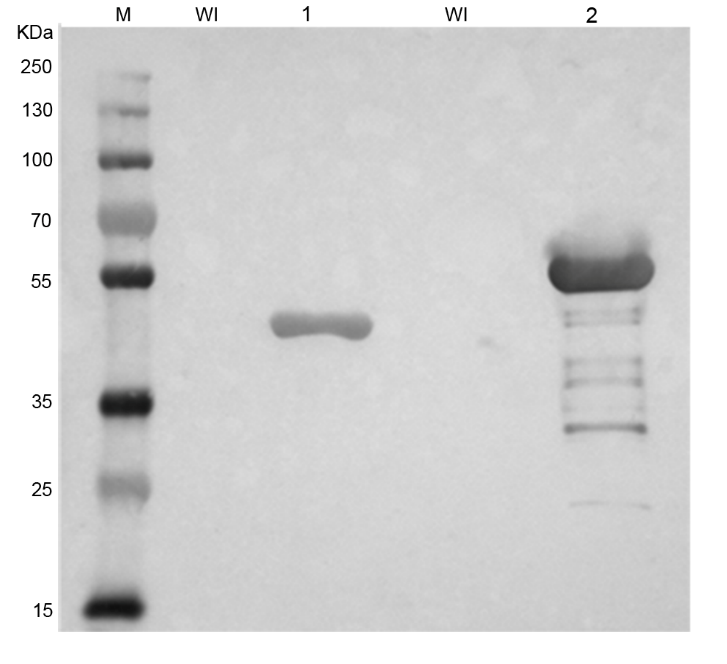


**Fig. S5 Western blot revealed using anti-His antibody**. Prestained protein marker (M), supernatant of cell lysates without induction (WI), second elution fraction of the purification of Xyl10C (lane 1) and Xyl10E (lane 2).


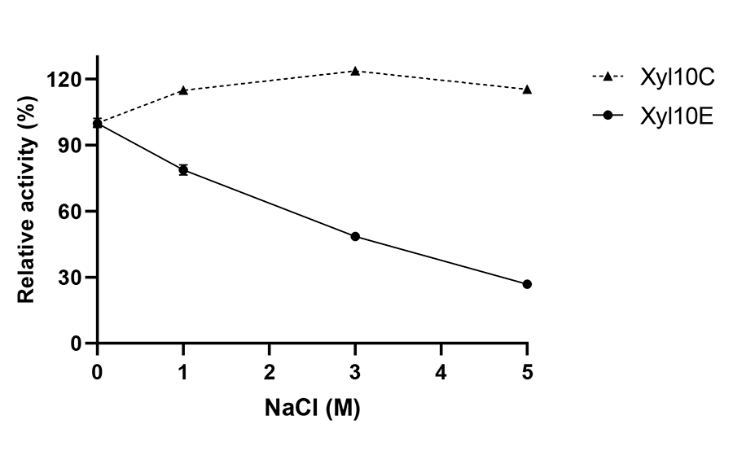


**Fig. S6** **Relative activity of Xyl10C and Xyl10E using increasing concentrations of NaCl.**


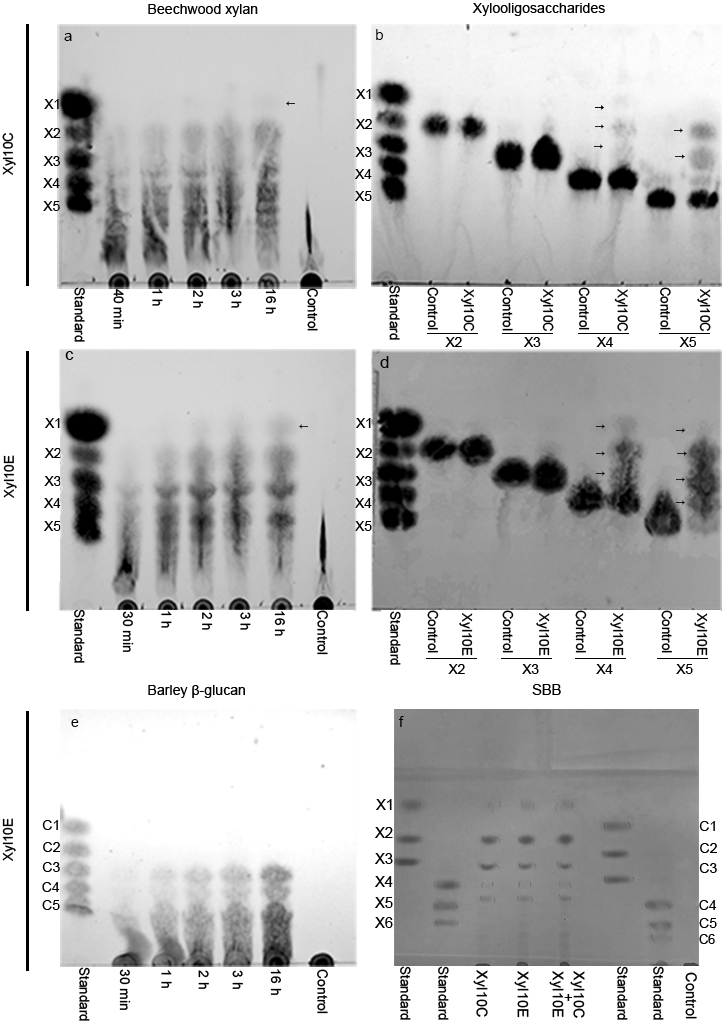


**Fig. S7 Thin layer chromatography analysis of hydrolysis products of Xyl10C and Xyl10E**. Time course degradation of beechwood xylan (1%) over different times for Xyl10C (a) and Xyl10E (c). Time course degradation of barley β-glucan (1%) over different times for Xyl10E (e). Hydrolysis patterns of the activity of Xyl10C and Xyl10E on short xylo-oligosaccharides as substrates (10 mM) at 60 min (b and d). Hydrolysis patterns of the activity of Xyl10C, Xyl10E and their combination on pretreated SBB (1%) as substrate (f). Control: enzyme without substrate, Standars; mix of xylose (X1), xylobiose (X2), xylotriose (X3), xylotetraose (X4) and xylopentaose (X5) 20 mM; and mix of glucose (C1), cellobiose (C2), cellotriose (C3), cellotetraose (C4), cellopentaose (C5) and cellohexaose (C6) 20mM.


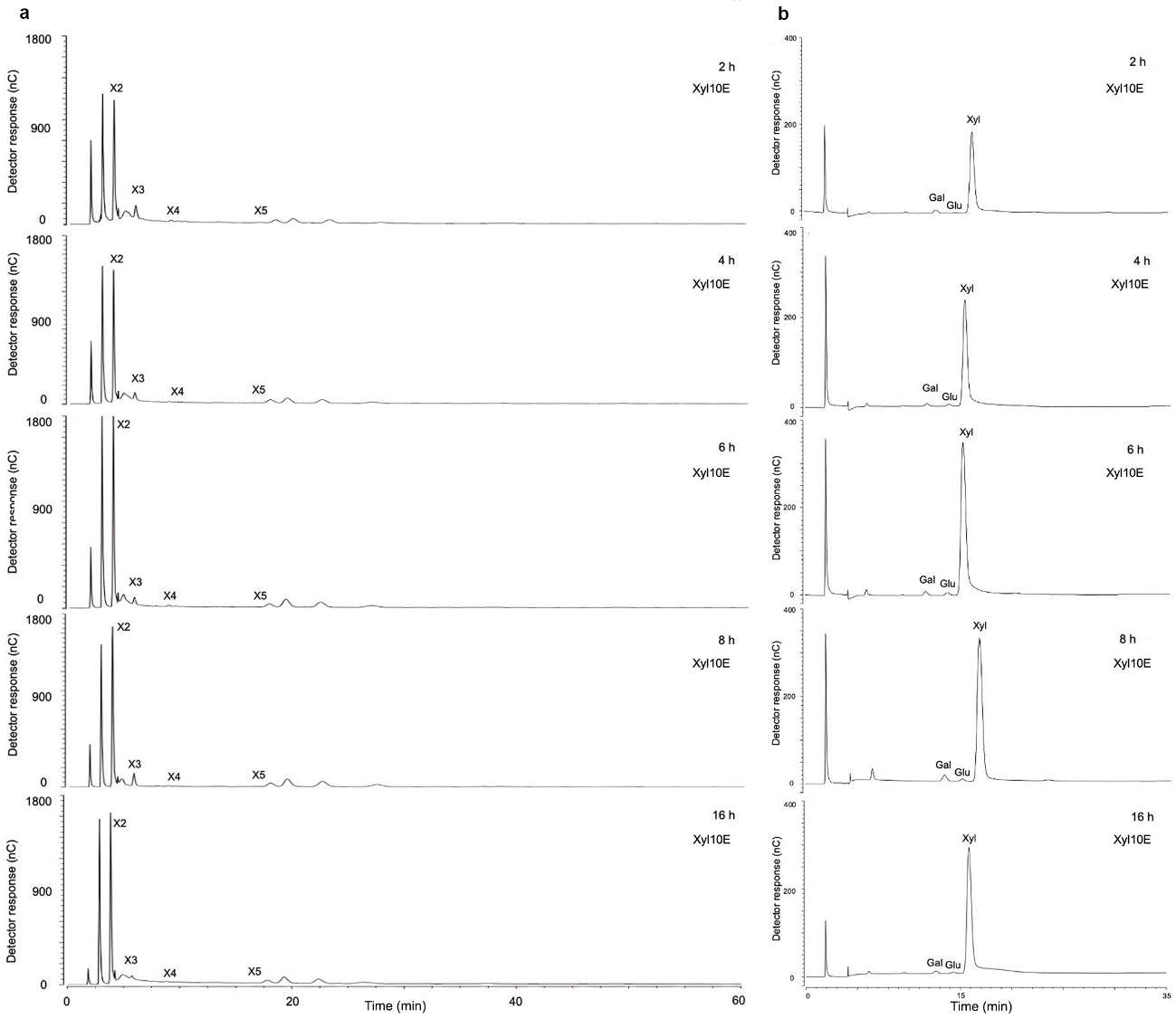


**Fig. S8 High performance liquid chromatography (HPLC) analysis of xylo-oligosaccharides (a) and monosaccharides (b) from time course degradation of pretreated SBB using Xyl10E.** Analysis of xylo-oligosaccharides (XOs)**.** Xylobiose (X2), xylotriose (X3), xylotetraose (X4) and xylopentaose (X5) as standards, sample dilution 1:500 (a). Analysis of monosaccharides Arabinose (Ara), Galactose (Gal), Glucose (Glu) and Xylose (X) as standards, sample dilution 1:10 (b).

**Table S1** The xylo-oligosaccharide (X2-X5) yield from 1% pretreated SBB

| **Reaction time (h)** | **Total XOs yield (%)** | **X2 yield (%)** | **X3 yield (%)** | **X4 yield (%)** | **X yield (%)** |
| --- | --- | --- | --- | --- | --- |
| 2 | 23.35 ± 0.11 | 14.35 ± 0.14 | 8.25 ± 0.19 | 0.76 ± 0.09 | 3.61 ± 0.09 |
| 4 | 22.92 ± 0.10 | 14.7 ± 0.11 | 5.8 ± 0.09 | 2.42 ± 0.08 | 5.02± 0.08 |
| 6 | 26.16 ± 0.06 | 18.62 ± 0.09 | 4.58 ± 0.08 | 2.96 ± 0.12 | 7.82 ± 0.07 |
| 8 | 21.40 ± 0.09 | 16.48 ± 0.10 | 4.28 ± 0.11 | 0.64 ± 0.11 | 6.99 ± 0.12 |
| 16 | 17.38 ± 0.08 | 15.34 ± 0.07 | 1.52 ± 0.12 | 0.52 ± 0.09 | 7.71 ± 0.18 |

| **Monosaccharides/ Xylo-oligosaccharides** |  |  | **Xyl10E** |  |  |
| --- | --- | --- | --- | --- | --- |
|  | **2 h** | **4 h** | **6 h** | **8 h** | **16 h** |
|  |  |  |  |  |  |
| Arabinose | 0.030 ± | 0.034 ± | 0.024 ± | 0.007 ± | 0.031 ± |
| Galactose | 0.010 ± | 0.013 ± | 0.014 ± | 0.021 ± | 0.010 ± |
| Glucose | 0.002 ± | 0.010 ± | 0.009 ± | 0.007 ± | 0.005 ± |
| Xylose | 0.287 ± | 0.400 ± | 0.622 ± | 0.556 ± | 0.613 ± |
| X2 | 1.140 ± | 1.168 ± | 1.480 ± | 1.310 ± | 1.219 ± |
| X3 | 0.656 ± | 0.461 ± | 0.364 ± | 0.340 ± | 0.121 ± |
| X4 | 0.060 ± | 0.192 ± | 0.236 ± | 0.051 ± | 0.041 ± |
| X5 | 0.0 | 0.0 | 0.0 | 0.0 | 0.0 |

**Table S2**. Production of monosaccharides and xylo-oligosaccharides after enzymatic hydrolysis Xyl10E of pretreated SBB. Values expressed in g L -1

**Table S3**. Comparative structural analysis of acidic amino acids related to halotolerance behavior of Xyl10C

| Overall comparison | | | | | Catalytic site environment comparison | | | | | |
| --- | --- | --- | --- | --- | --- | --- | --- | --- | --- | --- |
|  |  | Hydrophobic residues (%) | Polar residues (%) | A/B ratio | Hydrophobic residues (%) | Basic residues (%) | Acid residues (%) | Polar residues (%) | Non polar residues (%) | A/B ratio |
| **Xyl10C** | All | 2.59 | 2.59 | 1.07 | 19.23 | 5.77 | 19.23 | 36.54 | 38.46 | 3.33 |
|  | Surface | 0.23 | 3.02 | 1.22 |  |  |  |  |  |  |
| **Xyl10B** | All | 2.65 | 2.65 | 1.05 | 19.64 | 7.14 | 21.43 | 33.93 | 37.50 | 3.00 |
|  | Surface | 0.27 | 3.78 | 0.86 |  |  |  |  |  |  |
| **Xyl10E** | All | 2.38 | 3.33 | 0.97 | 23.64 | 9.09 | 14.55 | 36.36 | 40.00 | 1.60 |
|  | Surface | 0 | 6.25 | 1.25 |  |  |  |  |  |  |

| **Enzyme/ligand** | **Xyloheptaose** | **Cellohexaose** |
| --- | --- | --- |
| **Xyl10C**  Common amino acids | Asn41, Asn132, Gln86, Gln225, Glu133, Glu262, His79, His227, Lys44, Trp83, Trp232, Trp322, Trp330, Tyr193. | Asn41, Asn137, Glu40, Glu133, Glu262, His227, Lys44, Trp83, Trp322, Trp330, Tyr193. |
| Differential amino acids | Glu274, Lys229. | Gln86, Glu274, Lys229, Ser270, Trp232, Tyr197. |
| **Xyl10E**  Common amino acids | Asn140, Asn41, Gln238, Gln90, Glu141, Glu275, His240, His83, Lys44, Trp245, Trp329, Trp337, Trp87, Tyr206. | Asn41, Asp145, Glu141, Glu275, Glu40, His240, Lys44, Trp329, Trp337, Trp87, Tyr206. |
| Differential amino acids | Tyr283. | Arg341, Asn140, Gln238, Ser207, Tyr283. |

**Table S4** Common and differential amino acids between Xyl10C and Xyl10E in relation to the ligand with LigPlot+
